# Power aware routing algorithms (PARA) in wireless mesh networks for emergency management

**DOI:** 10.1371/journal.pone.0204751

**Published:** 2018-10-15

**Authors:** Tawfik Al-Hadhrami, Faisal Saeed, Funminiyi Olajide

**Affiliations:** 1 School of Science and Technology, Nottingham Trent University, Nottingham, United Kingdom; 2 College of Computer Science and Engineering, Taibah University, Medina, Saudi Arabia; Beijing University of Posts and Telecommunications, CHINA

## Abstract

Wireless Mesh Networks (WMNs) integrate the advantages of WLANs and mobile Ad Hoc networks, which have become the key techniques of next-generation wireless networks in the context of emergency recovery. Wireless Mesh Networks (WMNs) are multi-hop wireless networks with instant deployment, self-healing, self-organization and self-configuration features. These capabilities make WMNs a promising technology for incident and emergency communication. An incident area network (IAN) needs a reliable and lively routing path during disaster recovery and emergency response operations when infrastructure-based communications and power resources have been destroyed and no routes are available. Power aware routing plays a significant role in WMNs, in order to provide continuous efficient emergency services. The existing power aware routing algorithms used in wireless networks cannot fully fit the characteristics of WMNs, to be used for emergency recovery. This paper proposes a power aware routing algorithm (PARA) for WMNs, which selects optimal paths to send packets, mainly based on the power level of next node along the path. This algorithm was implemented and tested in a proven simulator. The analytic results show that the proposed power node-type aware routing algorithm metric can clearly improve the network performance by reducing the network overheads and maintaining a high delivery ratio with low latency.

## Background

An important factor in natural disasters and emergency situations is the availability of power resources. The aftermath of terrorist attacks in London and the natural disaster of the Japanese tsunami, clearly demonstrated the limitations of current responder communication technology “[1–2]”. The absence of power absence in such situations is fatal “[2–3]”. However, countervailing power resources can be used in such communication (i.e. wireless routers or gateways powered by vehicular batteries). Wireless mesh networks (WMNs) have emerged as an attractive networking paradigm for instant and cost-efficient communications. Disruption-tolerant algorithms have been proposed to be employed for emergency/disaster management “[3–6]”.

WMNs offer many benefits, such as ambient power and low operational cost while maintaining flexible deployment, good coverage and robustness “[7–8]”. Such features mean WMNs are suited to provide excellent services in incident situations. Moreover, their capabilities contribute well in recovering from network failure due to their auto-configuration, self-organization, and self-healing characteristics. A number of research questions have been raised regarding mesh nodes’ power levels “[9]”, stable path selection “[6]”, safe backup routing and optimal paths with power stability. Furthermore, several future research issues have been identified, including their throughput, capacity, scalability, security, traffic congestion in smart cities “[10]” network integration “[11]” and their use in public safety communication “[12]”.

**Fig 1:**
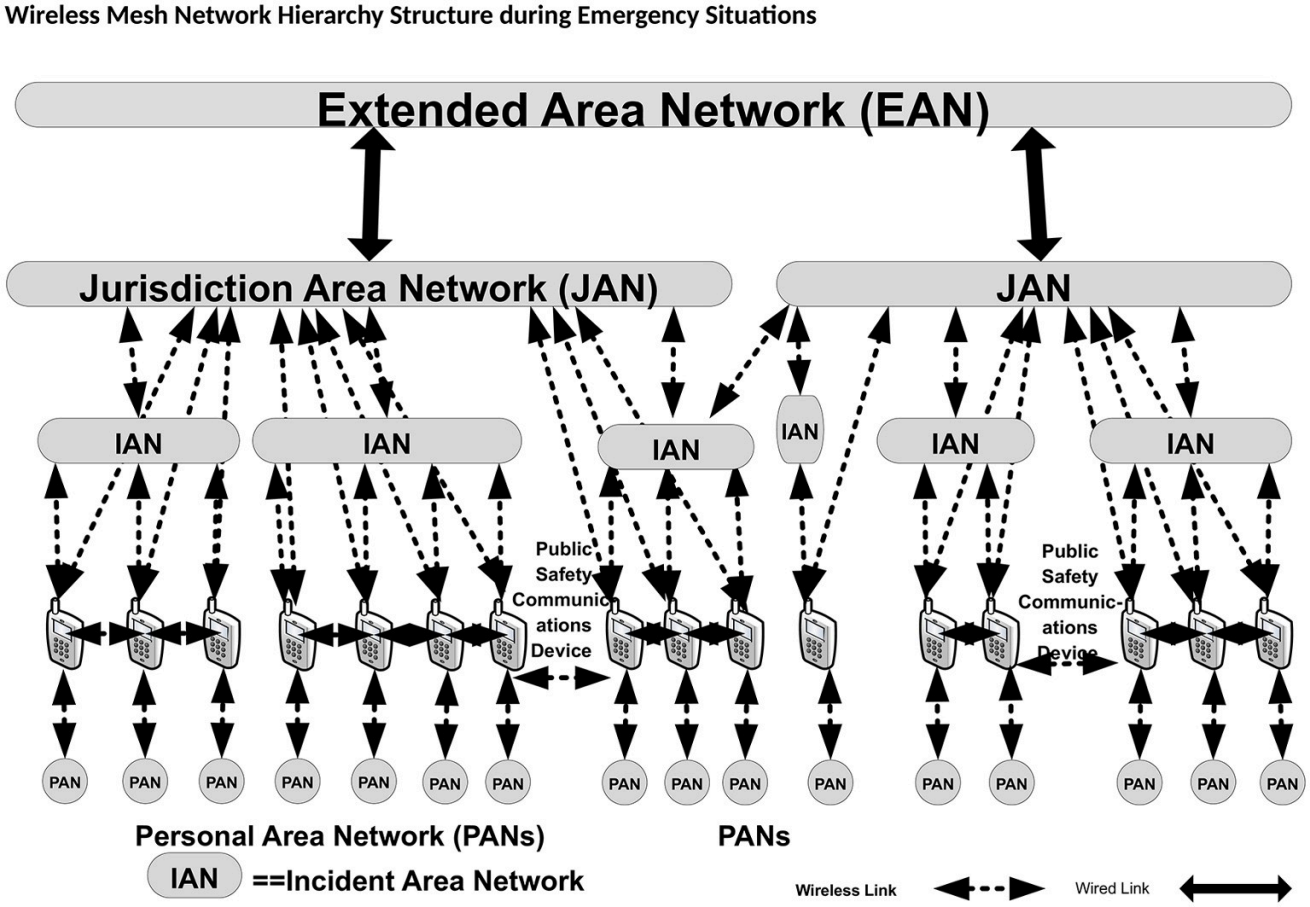
The network hierarchy structure for public safety communication devices (PSCDs) “[12]”.

Four types of communication networks for public safety communication are defined by the SAFECOM report “[12]”. “Fig 1” is a conceptual diagram showing these four types of networks and their interrelationships, categorized based on the service level. Personal area networks (PANs), are composed of various types of telecommunication end-user equipment and short-range communication devices such as wireless sensors (to be used for Internet of Things “[13]”), spread close to the objects of interest. Incident area networks (IANs), are typically a collection of WMNs that can communicate with each other through wireless links, thus allowing inter-IAN communications. Jurisdiction area networks (JANs), are the main telecommunication networks for data traffic that is not managed by the IANs. These consist of fixed infrastructures used for routine emergencies by police, firefighters and responders (this is the case of the European TETRA system) and an Extended Area Network (EAN), is any geographical network that acts as a backbone for JANs. It can be used to connect different JANs to each other and to provide connection to the head office.

Power consumption is the major issue in wireless networks “[12]”. During the life of the disaster-response network, power is essential and its loss is fatal. Therefore, minimizing power consumption per message is a key performance objective of such networks, in order to prolong the lifetime of a battery-operated device until the infrastructure is restored “[14]”. Thus, it is necessary to prolong the lifetime of the channels to transmit a whole package of data. A number of researchers have considered the power metric as stand-alone metric to be used for multi-hop mesh networks “[14–16]”: in other words, enhancing the routing performance of the WMN must be linked with the energy status. In this paper, a power node-type aware routing algorithm for disaster-response networks is proposed to explore the nodes’ power utilization. By knowing the node’s power, the designed algorithm will be able to include or exclude a mesh node in the path to the final destination. Thus, the proposed algorithm aims to enhance the routing between mesh nodes by using a power-aware routing scheme. We have investigated the routing selection based on power utilized and the performance power algorithm in various scenarios. This work has considered the amount of power in a channel and the particular type of channel that will guarantee the streaming/sending information without any power loss or fading, which is a critical factor during emergency situations and natural disasters. A number of proposed power algorithms have neglected to consider important factors such as channel type “[9–16]”. This work has considered the situation where numerous mobile or static rescuers, organized in teams, are distributed geographically in the emergency area, and undertake communications with the emergency headquarters or end-to-end, based on the public safety communication networks standard, as depicted in “Fig 1”. The results show that the proposed power aware routing algorithm (PARA) significantly enhances the incident area network in terms of routing overheads, packet delivery ratio and latency.

The remainder of the paper is organized as follows. In section 2, the related work on power routing algorithms in WMNs is reviewed. Section 3 describes the proposed power-aware routing algorithm and explains our model implementation of the algorithm based on the structure of public safety device communication. Section 4 presents the results of simulations in various scenarios and discusses the quality of service (QoS) and power consumption. Finally, section 5 presents conclusions and suggestions for future work.

## Related work

A number of routing algorithms have been designed for WMNs in recent years “[15–16]” and the issue of power consumption has been tackled in number of ways. The power algorithms described in “[17–18]” attempt to find the optimal path with minimum energy consumption and are mostly based on ad-hoc routing protocols, which have been designed for highly dynamic mobile networks (i.e. ad-hoc networks). All these routing protocols typically use the hop-count as their routing metric. However, the hop-count metric is not able to establish paths with maximum throughput in hybrid or infrastructure mesh network “[19]”. Thus, non-optimal throughput paths are established, due to the fact hop count does not differentiate between high quality and low quality wireless links.

The Expected Transmission Count (ETX) metric is proposed in “[20]”. ETX is a measure of link quality which considers the predicted number of times data packets need to be transmitted and re-transmitted at the MAC layer to successfully traverse a link. The ETX metric for a path is the aggregation of the ETX values of the individual links of the path. The forward delivery ratio of a link is defined by *d*_*f*_, while d_r_ is the corresponding parameter for the reverse direction of the link and d_f_ x d_r_ can be read as the probability of successfully transmitting a data frame. ETX is defined as follows:

$$\text{ETX}=\frac{1}{d_{f}\times d_{r}}$$ (1)

where the significant shortcoming of ETX is that it does not consider the transmission rate of links, which is the amount of data that can be sent between nodes. Thus the packet’s power is not considered in the ETX.

The Expected Transmission Time (ETT) metric proposed in “[21]” is an extension of ETX to overcome its shortcoming with respect to the transmission rate of links. ETT is defined as follows:

$$\text{ETT}=\text{ETX}\times \frac{S}{B}$$ (2)

where S represents the packet size and B represents the bandwidth or capacity of the link. However, the ETT has significant limitations, when applied to multi-radio networks.

The Weighted Cumulative ETT (WCETT) metric “[18]” is designed to overcome the key limitation of ETT in the context of multi-radio mesh networks, As it is unable to deal with the multi-radio nodes, ETT is not able establish channel-diverse paths that exploit the availability of multiple inter-node links. This can be achieved by involving the channel diversity in the metric.

The WCETT metric of the path p is defined as follows:

$$WCETT_{p}=\left(1-\propto \right)\times \sum_{i\in p}^{}ETT_{i}+\propto \times {\text{max}}_{i\leq j\leq k}X_{j}$$ (3)

*Xj* expresses the aggregate values of ETT links on channel *j*. The first term of the equation, accumulates the individual links of ETTs and nominates the shorter, high quality paths. The second term of the equation adds up the ETT’s links and chooses the maximum over all channels. α is a tuneable parameter within the range 0≤α≤1. The WCETT does not support channel reuse, which is considered as a limitation. Moreover, WCETT, ETT and ETX still do not consider link life, link congestion, link power, neighbour node power or interference that will affect the route in reaching its final destination.

The cooperative routing algorithm in “[22]” takes the active electronic power consumption into consideration when constructing the minimum power route from source to destination. However, prolonging the life of the route is not considered, which is a very important factor in such cases, as disaster and emergency services need to keep the cooperative path active for a long time.

Power conservation is one of the main objectives of routing algorithms for many types of wireless networks, such as mobile ad-hoc networks, sensor networks and mesh networks “[22]”. However most of the existing cooperative-based algorithms are implemented by finding the shortest route to the destination first and then improving the route using cooperative communication. Thus, it is important to try tackle both these issues at the same time.

The minimum power cooperative routing algorithm (MPCR) “[23]” is designed to construct a minimum power route that guarantees throughput from source to destination through the optimal route, regardless of whether it is the shortest path or not. However, the MPCR’s shortcoming is that the life of that optimal route to a certain destination may be short, because of problems such as breakdown of power resources along the path.

The adaptive power-aware routing algorithm “[16–17]” extends the power saving routing (PSR) algorithms in “[18]”. PSR is implemented by including a fading coefficient in the event where a station needs to make a decision to use next hop. This decision is based on the minimum power consumption with respect to the real channel conditions, such as multipath fading. However, although the extended PSR algorithm is suitable for normal network situations, it is not suitable in cases where hybrid mesh networks are working in incident area networks (IAN).

In “[24]”, idle radio observation is proposed in energy efficient channel assignment and routing algorithm-traffic demand (*E*^2^ CARA-TD), which is implemented by turning off a number of radio interfaces, if the performance of the network is not impaired. However, it does not consider prolonging the life of the route for that interface with neighbour nodes.

A power aware cooperative routing (PACR) algorithm is proposed in “[25]” for wireless mesh networks (WMNs). The optimal route from the source to destination in this algorithm is targeted by taking into consideration the power consumption of the cooperative communication. However, this was implemented without considering a minimum lifetime for this route. In addition, the PACR is using the next hop as a metric to find the optimal route; this method is not able to establish paths with maximum throughput in hybrid or infrastructure mesh networks. However, the power-node-type aware routing algorithm has tended to be used on ETX as a routing metric to overcome these gaps.

Parameters identification of chaotic systems via chaotic ant swarm “[26]” is proposed for solving optimization problems in communication technology, and this should be considered in the new intelligent system.

Particle swarm optimizer with crossover operation (PSOCO) “[27]” is proposed as an optimization scheme which adopts two different crossover operations. This algorithm will be employed in our new work, which is based on the Wireless Mesh Network and Internet of Things (IoT), for better quality communication among the emergency services. The advantage of PSOCO is its ability to find the optimal node in a multi-layer network infrastructure; this advantage will be employed in the new promising technology “The Internet of Things (IoT),” due the layout of the IoT’s infrastructure layers. However, the APRA considers the optimal route in different layers of the wireless mesh network.

This work will overcome the above-mentioned limitations by using a power metric to find the optimal path in a multi-hop mesh network. There are no approaches specifically investigating the problems associated with path selection and the routing of packets based on power level of neighbour’s nodes in the context of emergency management. This is important to address because, in the absence of power, there is no disaster response-network.

## Power aware routing algorithm

The Power Aware Routing Algorithm (PARA) presents a practical route channel selection, which aims to improve the routing between wireless mesh nodes in the emergency-response-network, based on the power utilized on a particular node. The PARA algorithm exploits information about different paths, when this is available on a particular node, to calculate the necessary power. Message delivery power probability helps to discover most reliable paths available, which are selected based on the neighbour node’s power level. There are three key challenges associated with opportunistic exploitation of paths with prolonged node power life and high quality of services (QoS). Firstly, the information must be maintained in routing tables. to measure the prolonged life route required and quantify the necessary power. Secondly, it aims to save power for each node by minimizing the overheads (retransmission, replication, and forwarding). Finally, it prioritizes the paths, based on the neighbour node type, either mesh router or mesh clients, with mesh router preferences.

To address the issues above, the first challenge, is that each node in the network must send a message advertising its level of power within a time interval, which is called *Power Level (PL)*. *S*ince nodes move in a finite space, under wide mobility assumptions, all packets will be delivered given an infinite amount of time. The second challenge is considering the number of copies adjusted for each message that sets bounds the number of actual copies of it that can be created. The third challenge involves a node type aware algorithm metric that prioritizes the next hop, either a mesh router or a mesh client, in which a mesh router is preferable. The above-mentioned advertising message is generated by using an adaptive routing OSPF protocol that generates messages such as link state and Hello packets.

### A. Open shortest path first (OSPF)

A power node-type aware approach to routing metrics could significantly improve the performance of both quality of service (QoS) and dynamic link state algorithms “[24]”. The Open Shortest Path First (*OSPF*) routing protocol is an adaptive routing protocol which has been widely researched and developed “[5]”. *OSPF* has two basic mechanisms to determine link state using a link state routing algorithm. The first mechanism involves Link State Advertisements *(LSAs)*, which are generated by each node carrying the status of its entire set of links, along with their costs. These advertising messages are flooded throughout the network. The second mechanism is Hello Packets *(HL)* which are used to determine if the link to a given neighbour is still alive by sending a Hello packet within interval window. Additionally, a dead interval, based on the Hello interval is used to detect dead links. The routes are reactively updated after the *LSAs* propagate through the network. With rapidly varying link quality, the only mechanism through which *OSPF* in its default configuration can detect link degradations is when four consecutive Hello packets are dropped. Since the size of the Hello packets is much smaller than that of data packets, a Bit Error Rate (BER) that results in four consecutive Hello drops will correspond to a significantly higher data packet drop rate. One mechanism that can be used to improve the performance of OSPF is a cost metric that is proportional to the BER of the link. However, this is a difficult proposition, given the lack of information exchange between the links that detect the optimal routes. In order to discover the optimal route, the power node-type aware metric is added to the OSPF routing protocol, in order to prolong route life, which improves the performance of the OSPF cost metric. Furthermore, several optimizations are performed on standard OSPF to serve efficiently in emergency situations, such as multi-link discovery, link selection based on congestion and channel diversity and local link repairing. The mesh network model considers the required optimization in incident area networks (IANs).

### B. Mesh network model

**Fig 2:**
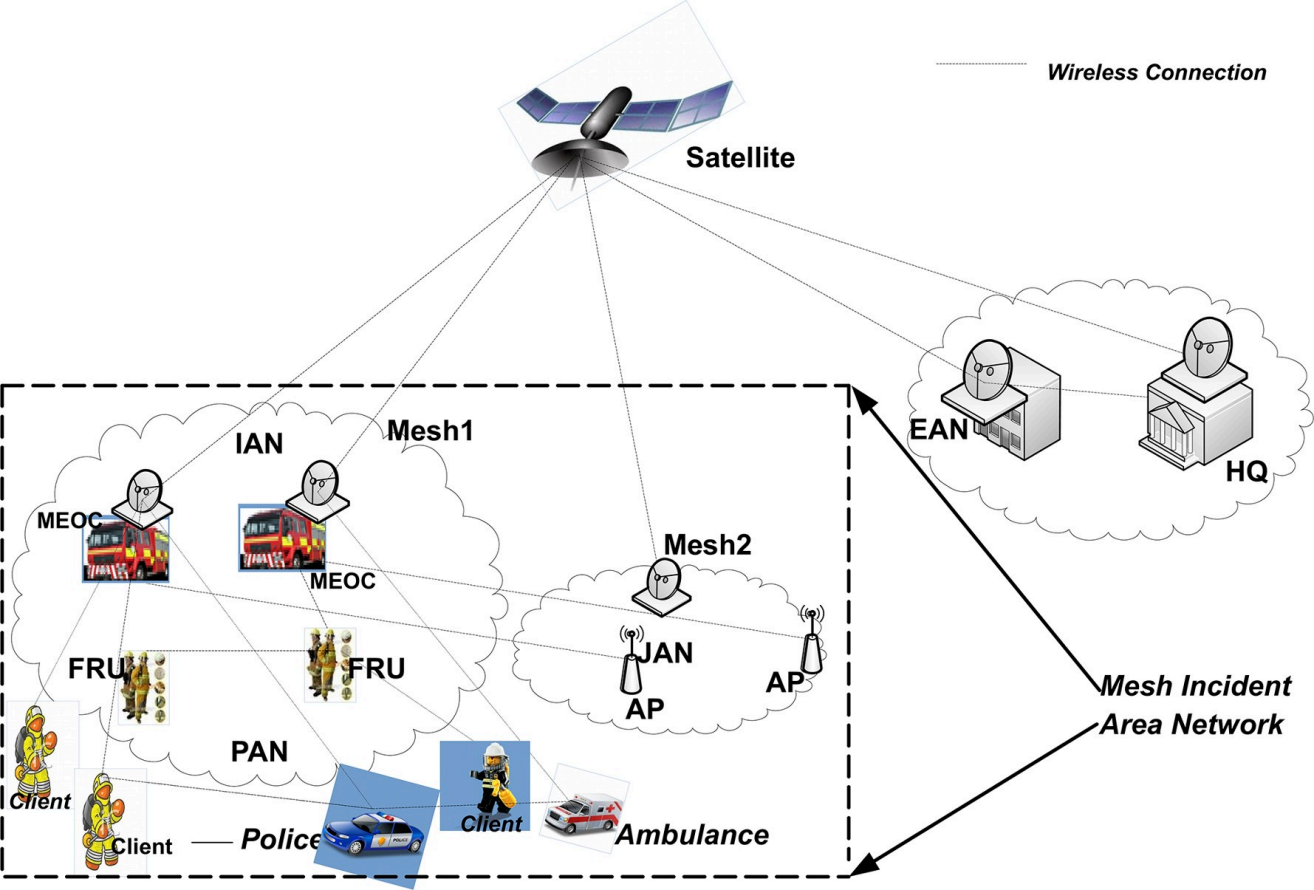
Wireless mesh network for incident area.

Consider a wireless mesh network consisting of a large number of mobile mesh routers and clients, where both routers and clients have a single omni-directional antenna “Fig 2”. The WMN part is working in the incident area to communicate with the head office of emergency management. It includes a power supplier, police officer, ambulance, operators and emergency agencies in the area.

**Fig 3:**
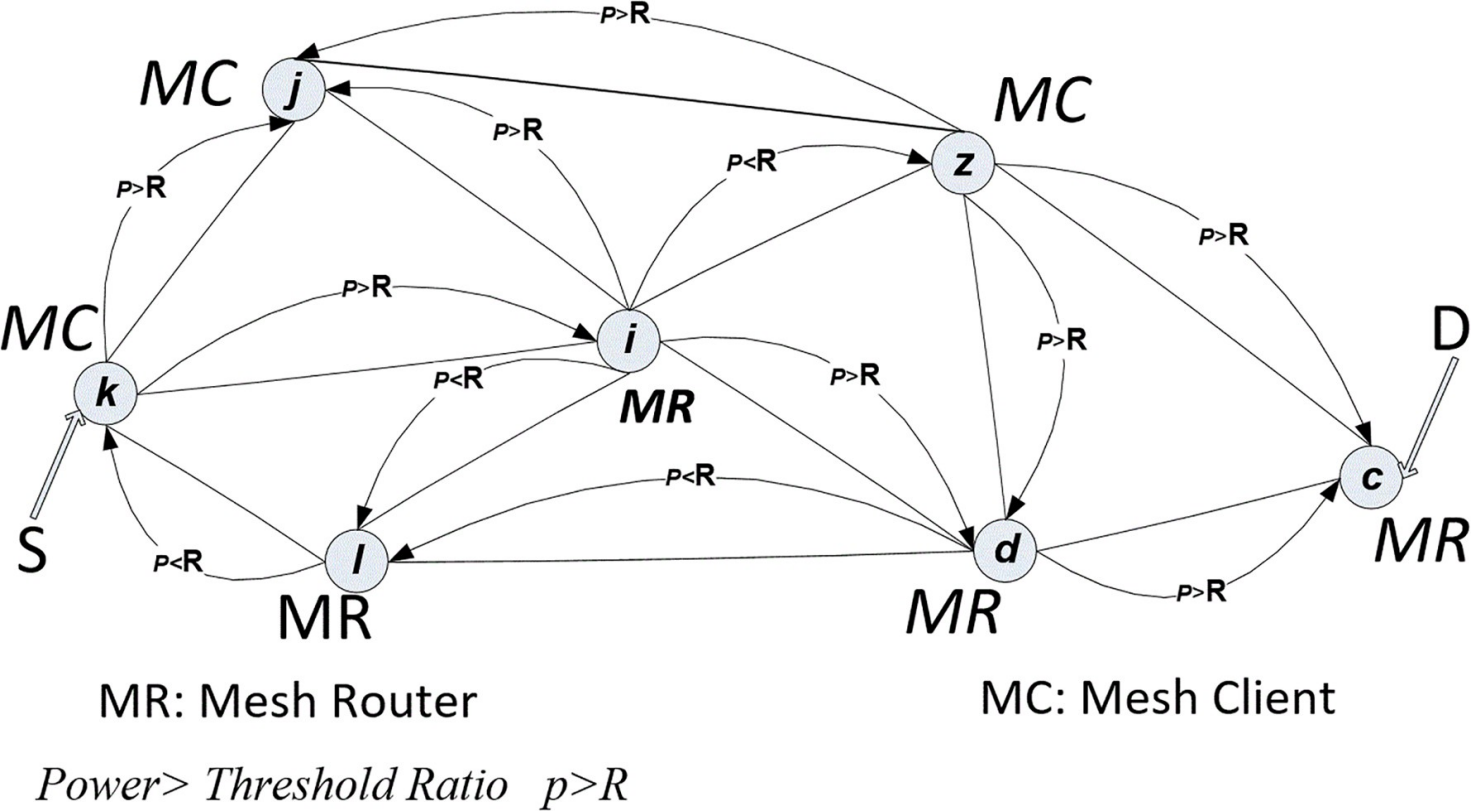
Mesh network power model.

The proposed mesh network model considered in this study is inspired by the idea of controlling the paths based on power. For instance, when node ***k*** is sending a packet along the path to neighbour node ***j***, the *Hello Packet* (*HL*) includes a *Power Level (PL)* banner, which informs the nodes about the current power level in a particular node within an interval window. For example, consider when a mesh *mobile client* ***k*** running in the incident area decides to communicate with head office emergency management c, as shown in the mesh model “Fig 3”, in order to send/receive visual video/images, maps, or voice information. However, the whole message package takes around 30 minutes, based on routing tables (*k-i-d-c*) Information is recorded regarding the power for each neighbour node in the emergency network. Thus, the routes are selected based on the power level of the nodes along the path (*k*, *i*, *d and c*), as explained in the next section. The nodes’ power must be sufficient according to a particular threshold ratio *R (p>R)*. The power ratio along the route must be enough to send a whole message package of information at one time without power interrupt or disruption. The optimal route is based on the value of the routing metric which is accumulated for the number of hops in the route to the final destination, as shown in Eq (4).

RouteMetric=∑{α×(Meshrouterpower*linkquality)×A+β×(Meshclientpower*linkquality)×B}α>β>0 (4)

**Fig 4:**
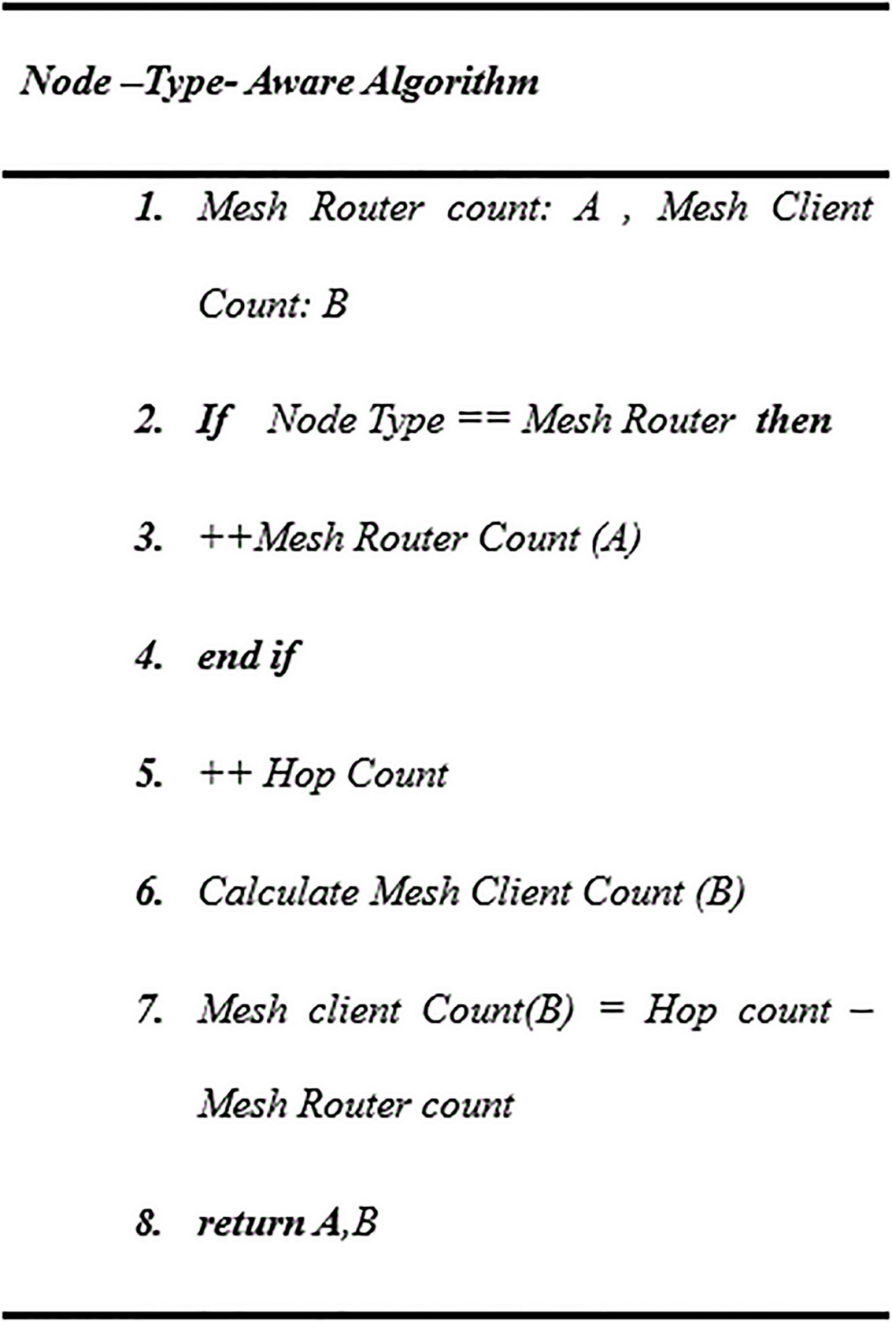
Pseudocode for node type aware.

The values of α and β are the weights associated with mesh routers and clients along the path; for this work α and β will be assumed as α = 4 and β = 1. The values of A and B are added to recognize the type and the number of mesh node (either router or client) that are called node-type-aware as the pseudocode explains in “Fig 4”.

**Fig 5:**
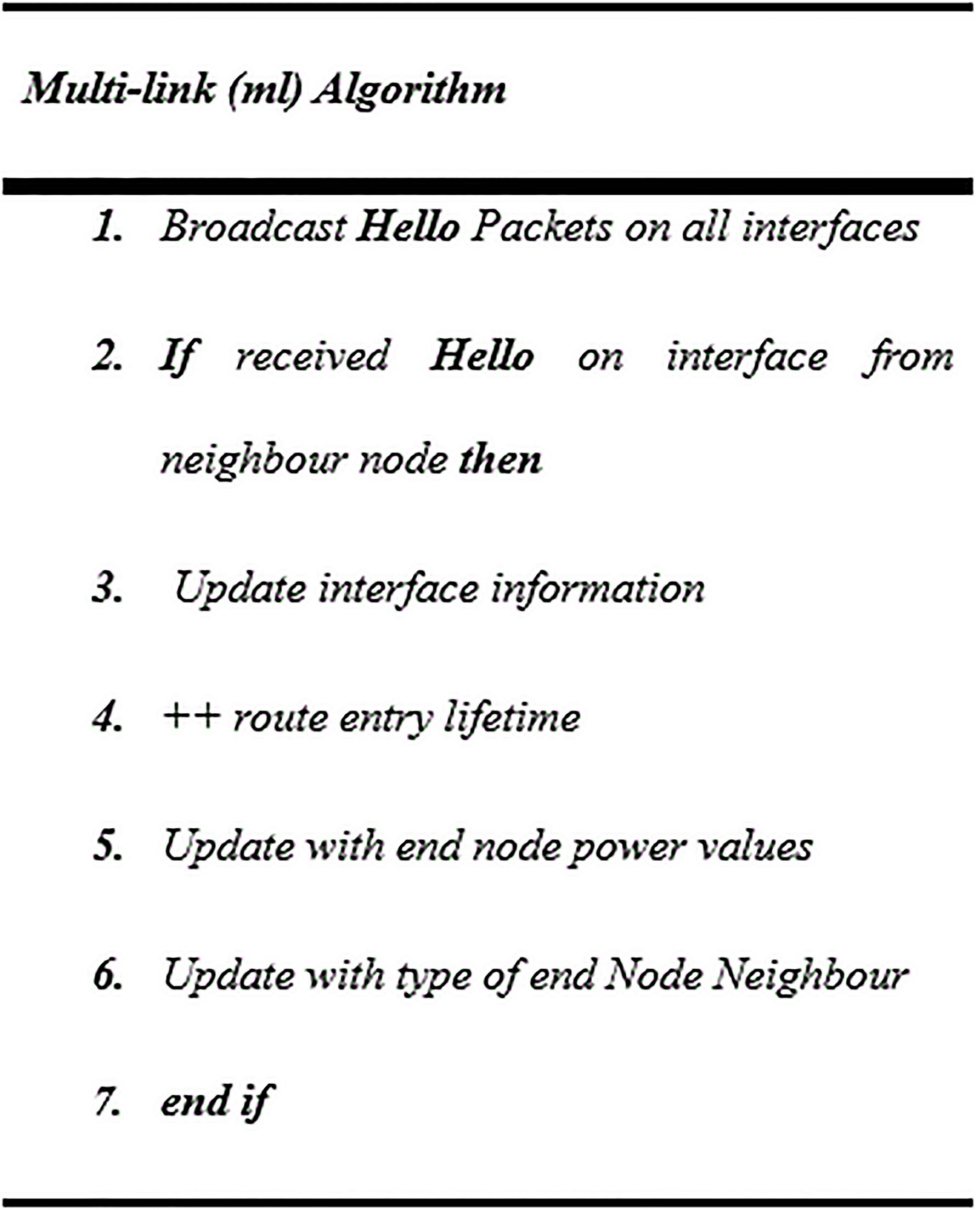
Pseudocode for multi-link mechanism.

For each node involved in the mesh network, a set of available links between node pairs are considered. This information is useful for local link repair and channel diversification mechanisms. Periodic *Hello* packets are broadcasted by each node at all interfaces, to determine the complete set of links available between nodes. If *Hello* packets are received from a node via a particular interface for the first time, a corresponding routing for routing entry is made, with an associated route lifetime value, as the pseudocode explains in “Fig 5”. The novelty of the power-type-aware routing algorithm lies in its ability to guarantee the streaming/sending information without any power loss or fading.

### C. The power routing algorithm

As observed above, incident networks are different from regular networks in that power is a significant factor in the lifetime of IAN’s connectivity, so the challenge is how to increase the lifetime for IANs and reduce power expenditure during an emergency. The emergency management team in the field controls the power between the nodes by recharging the mobile station’s battery. Battery operators provide mobile stations with power in such cases of emergency and natural disasters. So only the nodes having the required power level can participate in route discovery and data transmission. Optimizing routes between network nodes based on the utilized power is considered in this algorithm. The power algorithm monitors the power level across the network and guides the route to the final destination.

The initial power for each node in the network is ***PL*** and the power consumption, represented by the receiving/transmitting, movement power and constant power, is referred to as (μ). Five basic parameters are considered

- Initial power assigned to the node *(PL)*, *Pl* = *Initial Power*
- Remaining Powe*r RP* = Σ (Initial Power -Power consumed per node). Power consumed per node includes Mesh Node Power = Size of Packets in Bits * Bit power
- Battery-life Status (BS) is the battery level based on the ratio, if (Battery Status ≤ 40%) Then set BS = 3, If (40% <Battery Status < 80%) Then Set BS = 2, If (Battery Status ≥ 80%) Then Set BS = 1
- Type of Data Transfer (TDT): Control packets or Data packets.
- Node Type Priority: the router or client mesh is considered; the mesh router is preferable.

For example, in “Fig 3” mesh client, *k*, decides to send a message to the mesh router in head office, *c*. The power node records the battery life status. Link-state Advertisement (LSA) sends a signal to check the neighbour information details, while *Hello* packets check the previous power level (*PL)* for the neighbour node and send it back to node *k*. In the first step, node *k* starts checking neighbour nodes’, which are *j*, *i*, and *l*, power. By checking node *j*, if it possesses enough power, its node power status is given the value 2 (based on the pseudocode for channel diversity), and it is a mesh_ client node type, so node *j* is blocked (recorded in table called *Nodes Blocked Table*). However, node *l* has less power, so its node power status is given value 3, although it is a mesh_ router type, so node *l* is blocked too. The power condition is given priority to be achieved. Thus, nodes *j and l* are blocked in step time one (*T1)*.

**Fig 6:**
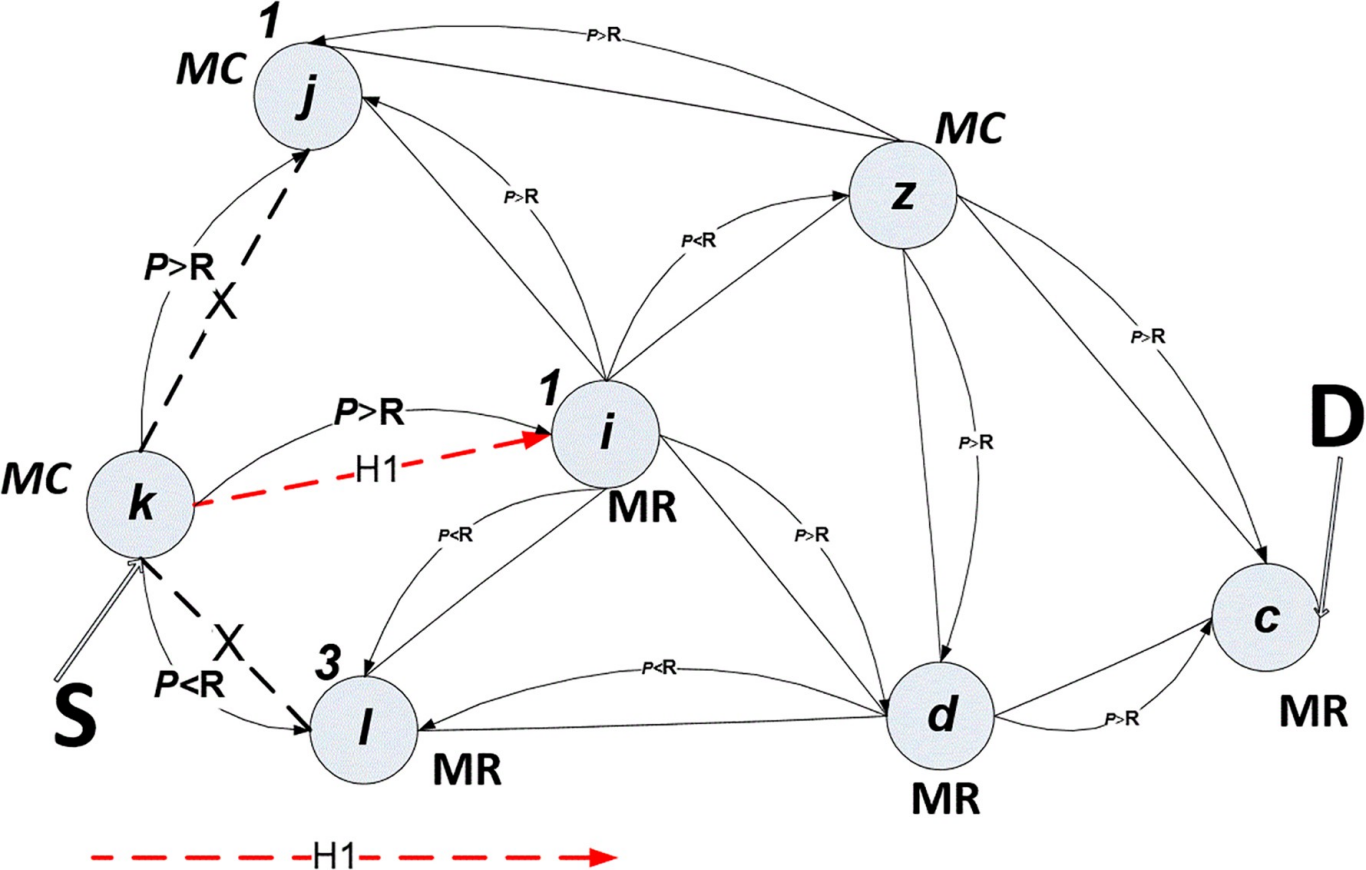
Power algorithm in time *T1*.

**Fig 7:**
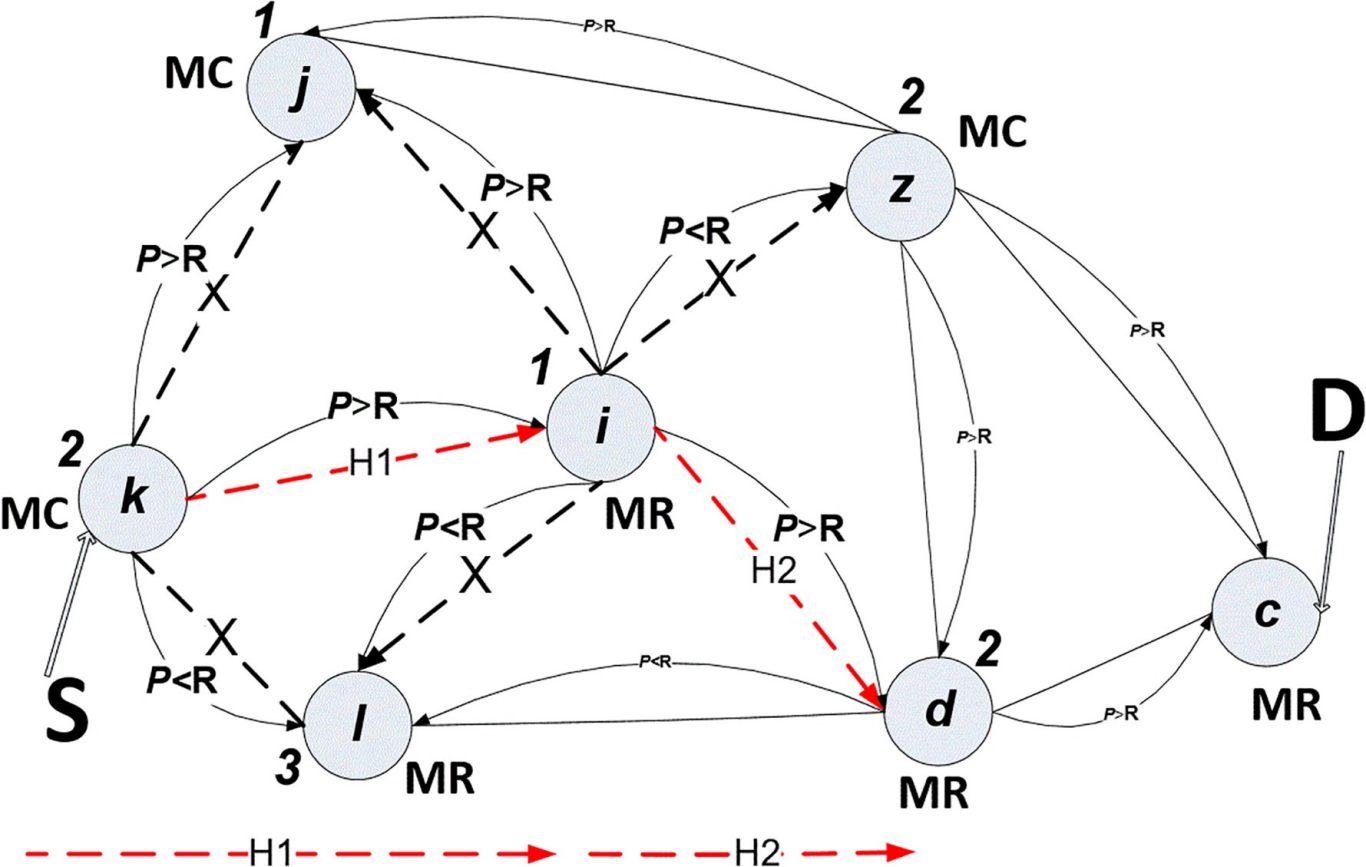
Power algorithm in time *T2*.

Node *i* possesses an excellent amount of power, so it has been given value 1, and it is a mesh router-type, so that node *i* is the next hop for the path to the final destination, as shown in “Fig 6”. Node *i* checks the power status of its neighbour nodes, which are *j*, *l*, *z* and *d*. Nodes *j* and *l* were blocked in the previous step, *T1*. The nodes *z* and *d* have both been given power status value 2, but *z* is blocked because it is a mesh_ client type, whereas node *d* is a mesh_ router type, which is preferable, as shown in “Fig 7”.

**Fig 8:**
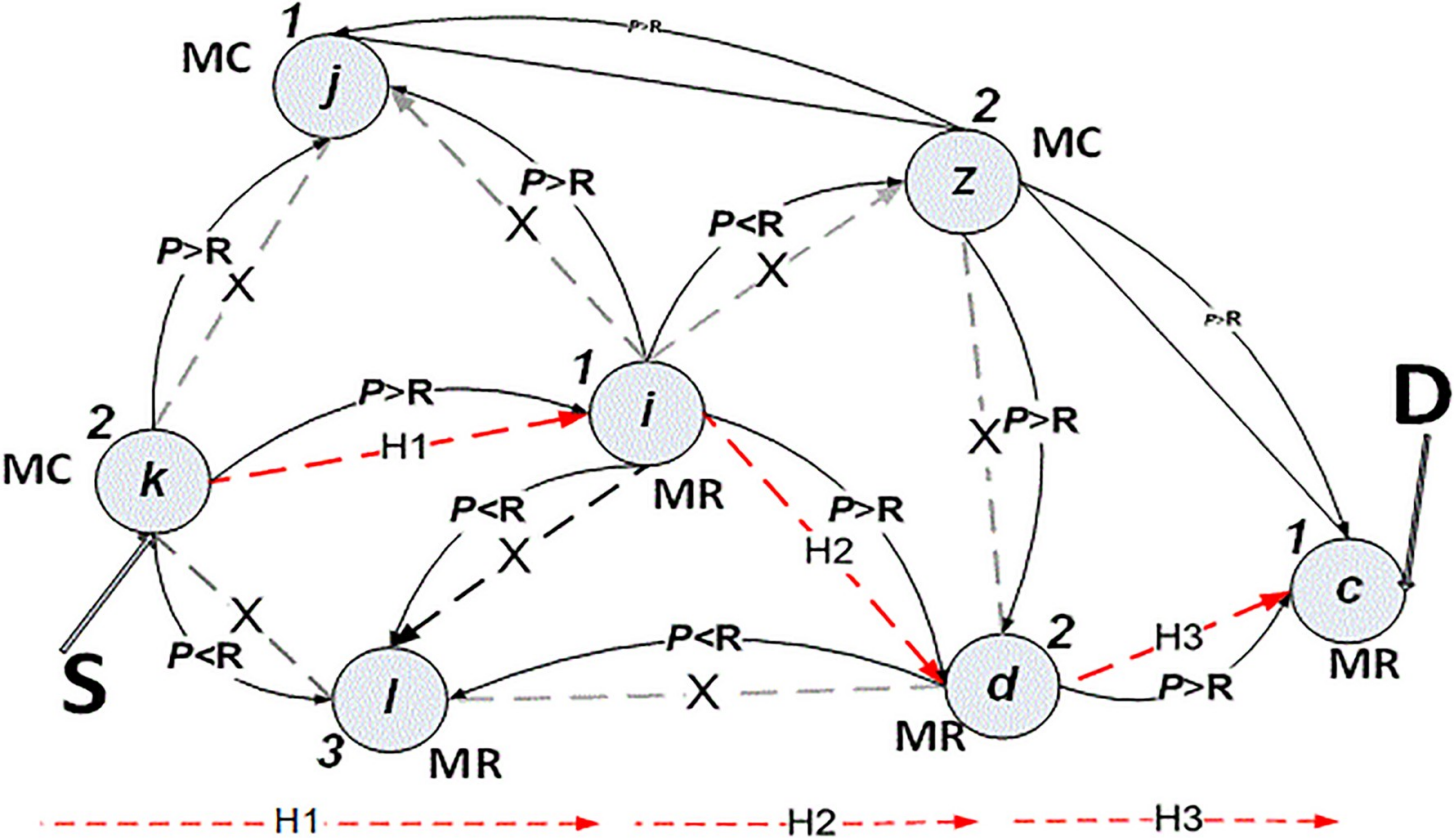
Power algorithm in time *T3*.

**Fig 9:**
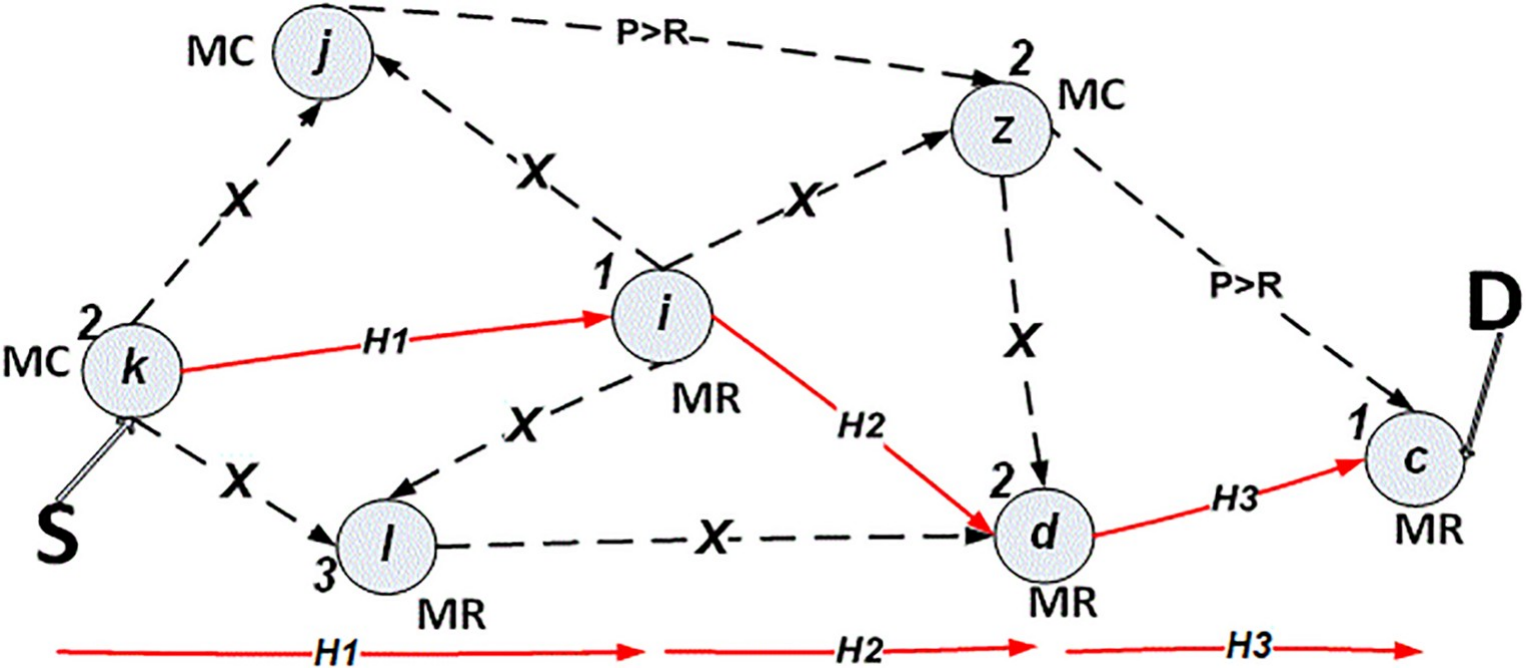
*S-D* path based on power algorithm.

Node *d* repeats the same process in order to discover its neighbour power nodes *z*, *l* and *c*. However, nodes *z* and *l* were blocked in *T2*. Thus, node *c* is the final destination. It has been given power value 1 and it is a mesh router, as shown in “Fig 8”. The entire path power guarantees communication without interruption or disruption, for a certain time as shown in “Fig 9”.

Loop back and infinite loop are serious problems in routing algorithms. This has been solved by marking the interface. These interfaces are stored in the *blocked table*, as shown in Table 1. The blocked table includes nodes which do not meet algorithm conditions, either in terms of power level or mesh type. The flag is using hop count as a sequence number, in other words, it is storing the reverse path to avoid infinite loop and use it for channel diversity.

**Table 1:** Next hop election mechanism.

S-to-D	*k-i-d-c*					BT
***Neighbour***	*j*	*i*	*l*			*l*, *j*
***k***	***×***	*√*	***×***			
***Neighbour***	*j*	*z*	*l*	*d*	*k*	*l*,*j*,*z*
***i***	***×***	***×***	***×***	*√*	*R-1*	
***Neighbour***	*i*	*z*	*l*	*c*		*l*,*j*,*z*
***d***	*R-2*	***×***	***×***	*√*		
***c***	*Final Destination*					

**Fig 10:**
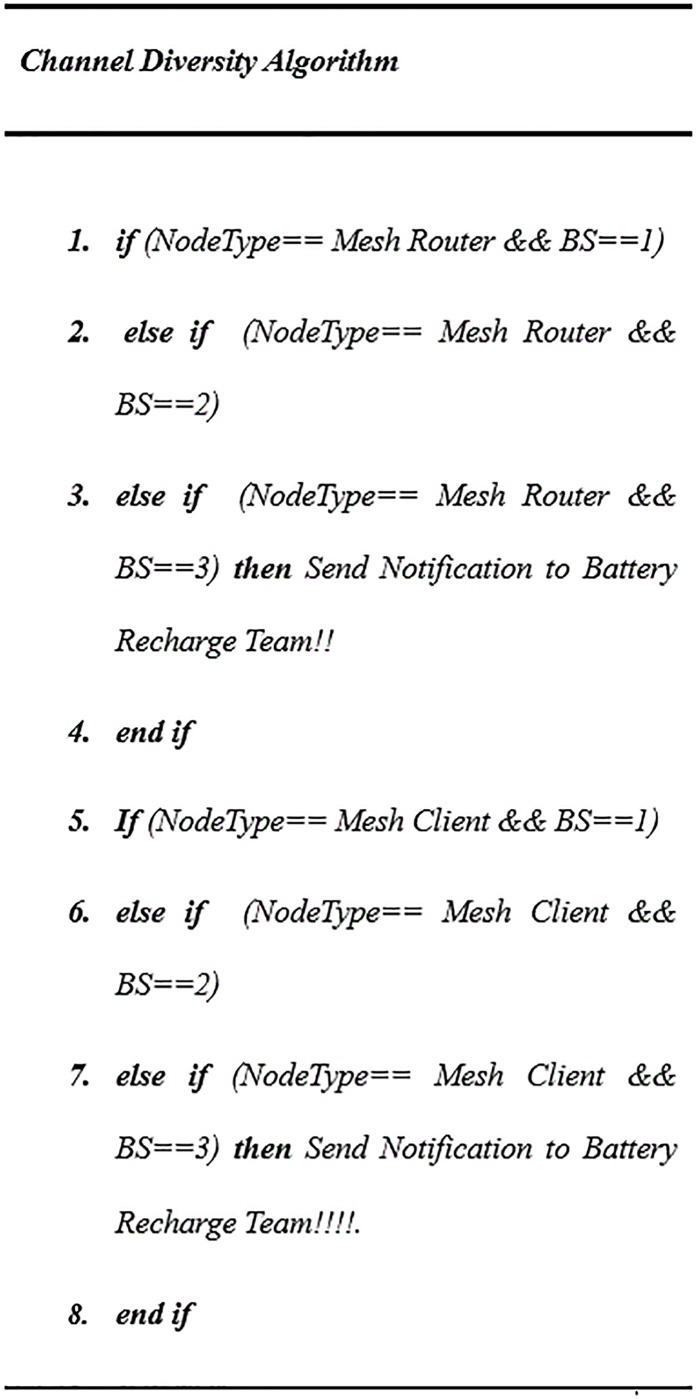
Pseudocode for channel diversity.

There are some special cases during route establishment, for example, if the node *i* had two or more neighbour nodes, such as *z* and *d*, which are mesh router type in the last *T2*, the second path preference will be held for redundancy of channel diversity. Channel diversity prioritizes the paths based on power level values (1, 2 and 3) and mesh node type (router or client) as explained in the pseudocode “Fig 10”.

The PARA routing metric *(RM)* is cumulative for quality of link multiplied by power for both types of mesh (router and clients) along the path. as defined in Eq (5) below:

RM=α×∑i=i+1n(ETX×1p)×A+β×∑i=i+1n(ETX×1p)×B (5)

**Fig 11:**
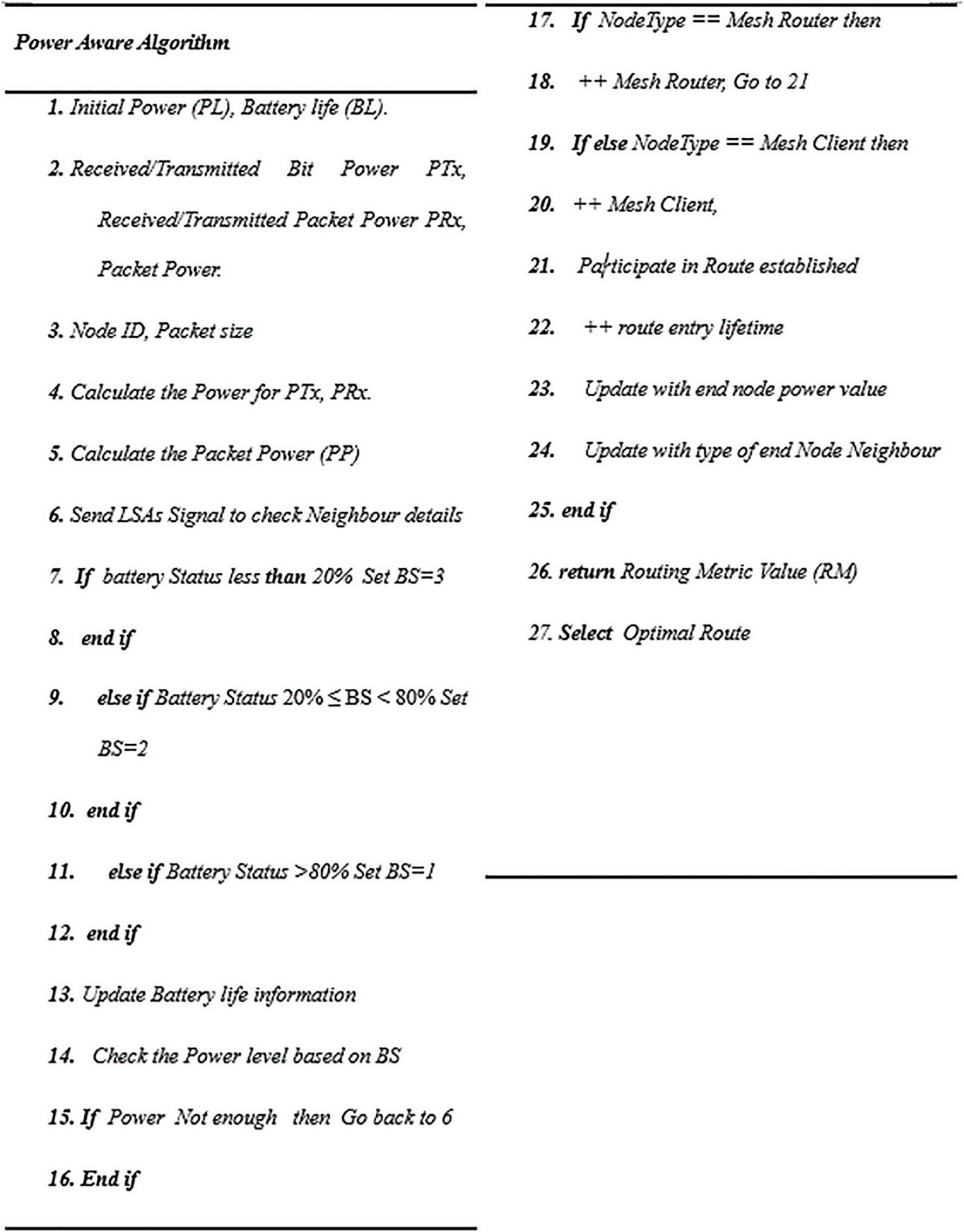
Pseudocode for power routing algorithm.

**Fig 12:**
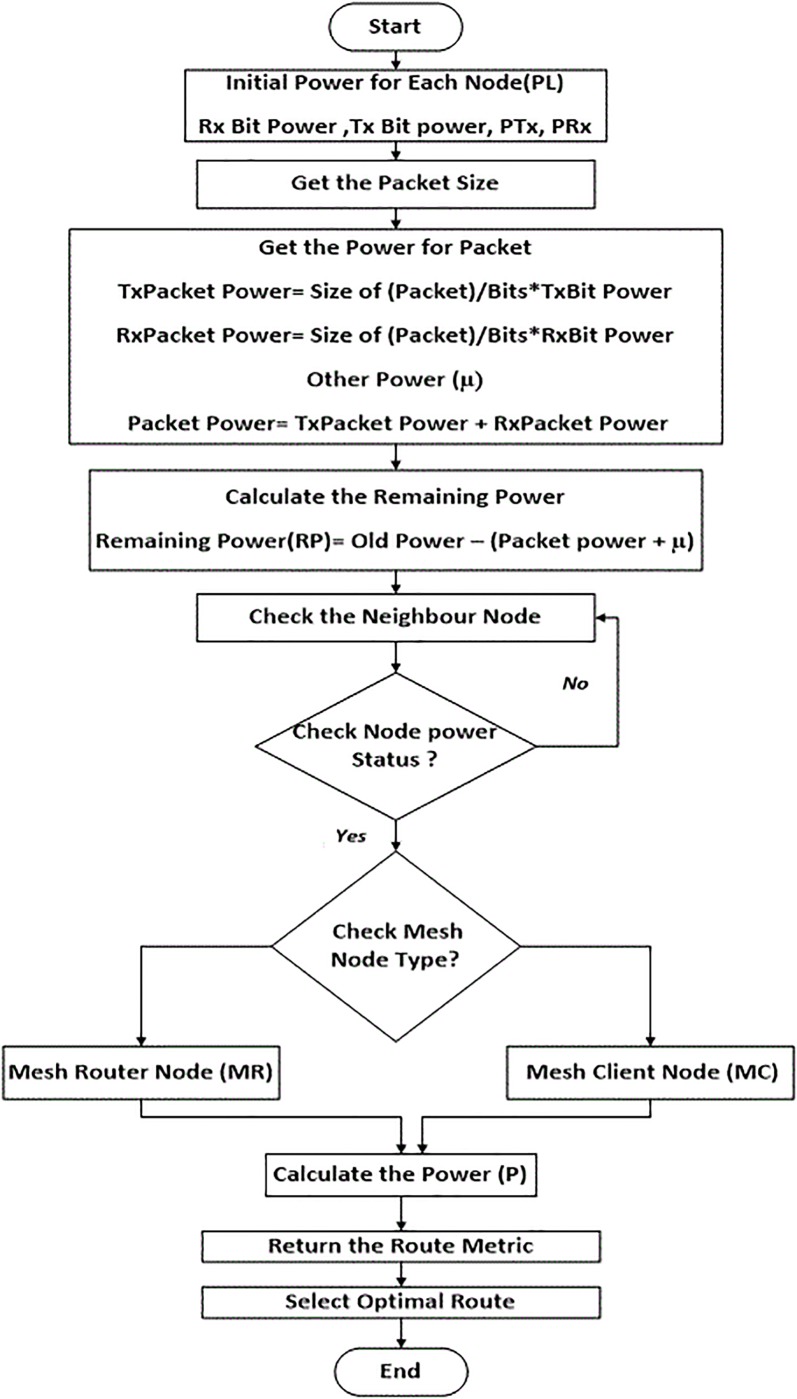
Flowchart for power algorithm.

The values α and β are the weights associated with mesh routers and mesh clients respectively. The current α and β values, α = 4 and β = 1, mean the weight of traversing a five-mesh router is equivalent to that traversing a single mesh client. The values of α and β were determined based on a large set of simulations. A and B are counted as the number of mesh routers and mesh clients, respectively, along the path. *EXT* is the link quality metric, as defined in Eq (1), and (**1**/***P***) is the reverse current amount of power for node *i*; the routing table stores the routing metric *(RM)* values and the number of mesh node types, either or router (*A*) client (*B*). These values are updated by LSA messages within the interval window. These variables’ values are then used to guide the path through the route in order to discover the optimal route. The optimal route is established when power level in the node is calculated and the mesh node type is recognized in the path. The smallest routing metric value *(RM)* is selected as optimal route value. These values are broadcasted by LSAs over the network within the interval window or if there is an instant change in the network topology, such as new mesh nodes becoming involved. The power aware algorithm using pseudocode is given in detail in “Figs 11 and 12”.

## Simulation results

### A. The NCTUns simulation environment

The NCTUns simulator “[28–29]” was employed in this study to implement and evaluate the Mesh Incident Area Network (MINA) for communications between the emergency management office (HO) and rescue team. The simulator has specific functionality within the Operating System (OS) that can capture packets generated by transmission application and route them to a simulation entity. The application uses the UNIX TCP/IP protocol stack on the machine where the simulator is installed, and then transmits packets to the tunnel interface network “[30]”. An application program can send or receive packets from the tunnel network interface with the same behaviour as a normal Ethernet network interface. The implementation, testing and evaluation in this work benefit from the advantages of the simulation environment, such as efficient power control, efficient generating and capturing video/image traffic, node mobility support, simultaneous running of multiple visual application programs, and Linux-based open-source software development. In this study, the Power Aware Routing Algorithm (PARA), SafeMesh (SM_AODV) based on AODV “[2]”, an energy-efficient scheme using AODV “[30]” and standard AODV were compared and evaluated.

The simulated network topology consists of 20 mesh routers (static and mobile) placed in regular 4×5 grid in a 1000 m × 1000 m area. In the simulation, the mesh router is equipped with five 802.11b radios tuned to the orthogonal channel. The network further consists of 10 mobile mesh clients, placed uniformly and randomly in the simulation area. The constant bit rate flows using the UDP protocol are established between uniformly selected source and destination mesh client pairs.

The following three scenarios were conducted to evaluate the performance of the PARA algorithm, under varying mobility and traffic load conditions, with different node configurations: Scenario 1: Varying the speeds of *Mesh_ Clients*; Scenario 2: Varying the number of *Mesh_ Routers*, and Scenario 3: Varying the speeds *Mesh_ Routers*. In the next section the power consumption in these three scenarios is measured and compared. The performance metrics are obtained by ensemble averaging the results 20 test runs. The default simulation parameters used in all the simulations are listed in Table 2.

**Table 2:** The parameters settings used in the simulations.

Parameter	Value
**Simulated Time (s)**	400
**Simulation Area (m)**	1000×1000
**Link Bandwidth (Mbps) Wireless**	11
**Propagation Model**	Two-ray ground
**Mobile Node Speed (m/s)**	1
**Max Mesh Client Speed (m/s)**	10
**Mobility Model for Mesh Client**	Random waypoint
**Packets Size (Bytes)**	512
**Transmission Mode**	Full-duplex
**Flow Rate (kbps)**	128
**Transmission range (m)**	250
**Transmission Power (dBm)**	15

### B. Performance metric

In each simulation, the following performance metrics are considered:

- Packet Delivery Ratio (PDR): the ratio between the number of data packets successfully received by destination and the total number of data packets sent by source nodes.
- Routing Packet Overhead (RPO): the ratio of control packets generated to the number of successfully received data packets.

Average Latency (AL): the mean time (in second) taken by data packet to reach to the their destination.

## Results and analysis

### • Scenario 1: Varying the speed of the *Mesh_Client*

**Fig 13:**
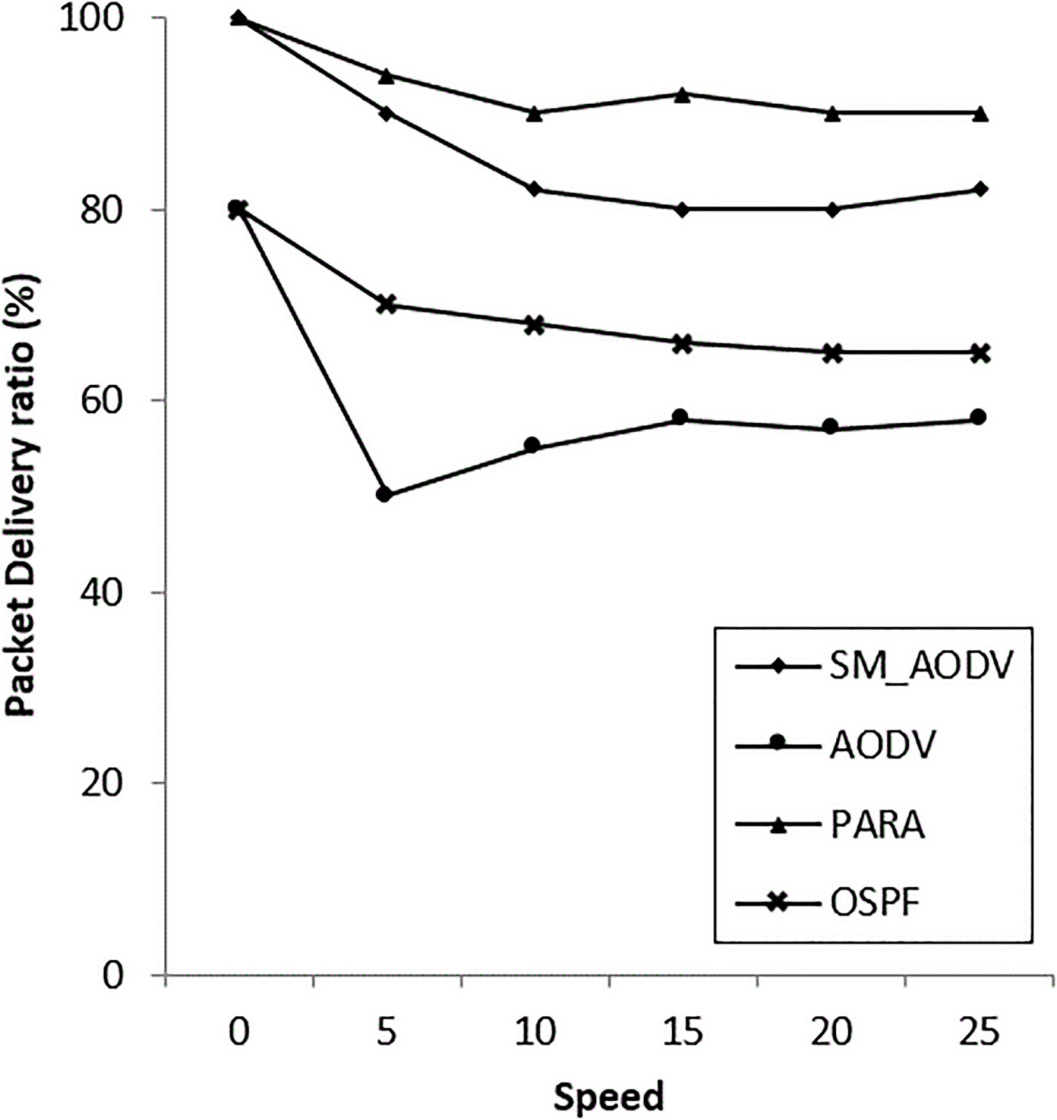
Varying *mesh_client* speeds with (PDR).

The speed of the mesh clients varied from 0 m/s to 25 m/s using the random waypoint mobility model is considered. The corresponding performance metrics for OSPF, SM_AODV and AODV are shown in “Fig 13”. To provide a reference for the range and variance, the results are shown with vertical bar showing the minimum and maximum values. The results indicate that at zero mesh client speed, AODV achieved a 77% Packet Delivery Ratio (PDR), while OSPF and SM_AODV achieved a PDR of almost 100%. However, the PDR declined rapidly as soon as the mesh client speed was increased. When the mesh client speed increased to a maximum of 25 m/s, the PDR of AODV dropped to 55%, and the PDR of SM_AODV fell to 80%, whereas the PDR of OSPF remained stable, fluctuating between 90% and 100%.

The network is reasonably congested, due to the high load and the fairly high node density. The routes created using AODV and SM_AODV suffered from significant performance degradation due to interference and congestion. Additionally, the SM_AODV routes were created based on mesh clients as intermediate forwarding nodes to final destination. This caused falls in PDR, because the mesh client capacity is less than that of the mesh router. The OSPF is exploiting the mesh routers’ capacity as intermediate forwarding nodes rather than the mesh clients. In other words, the mesh router is preferable in case of the OSPF. As a consequence, both SM_AODV and AODV had significantly increased routing overheads.

**Fig 14:**
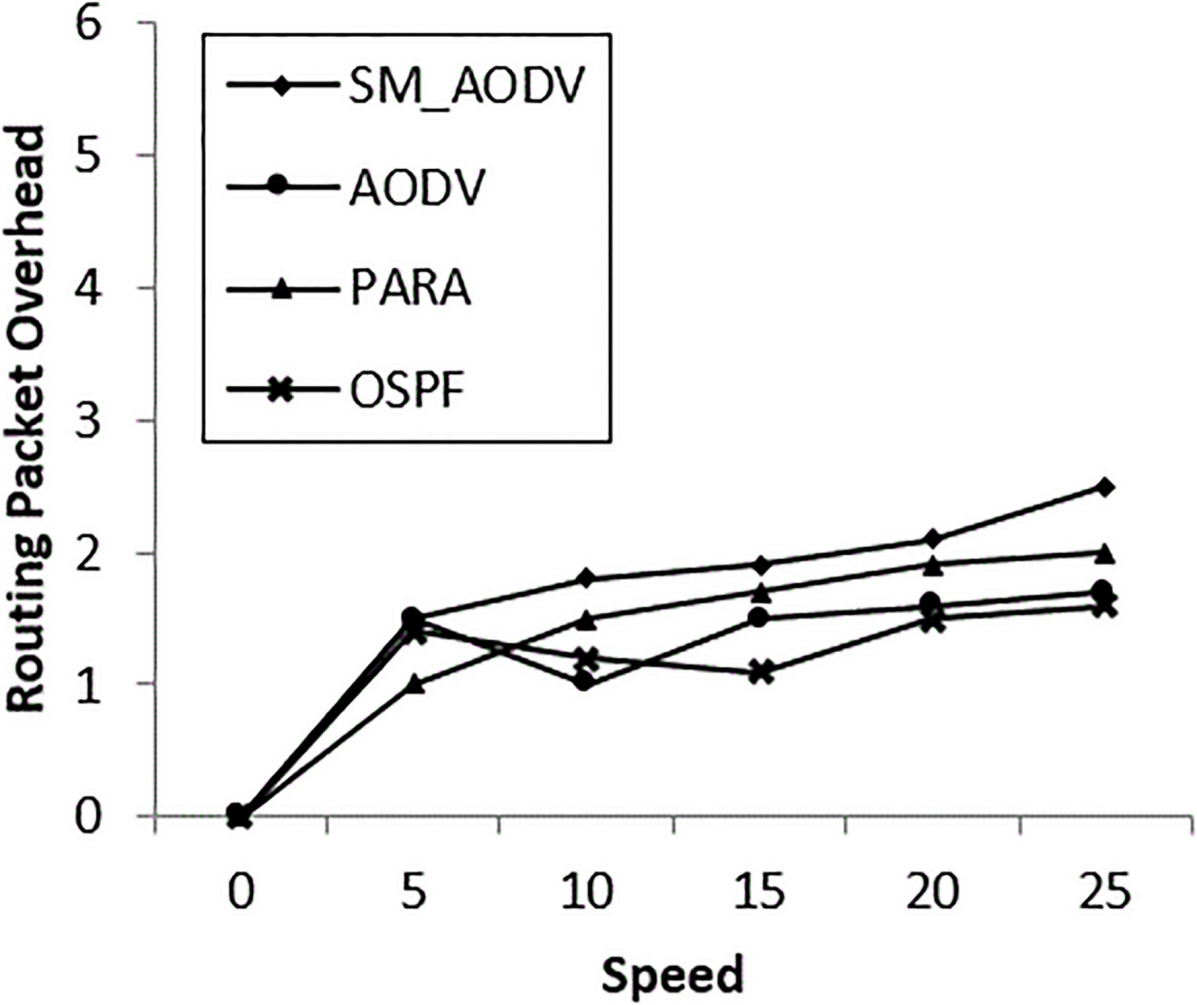
Varying *mesh_client* speeds with (RPO).

AODV does not take into account the congestion and load by periodically measuring the ETX. However, the computation of the ETX metric does not provide fast enough coverage at higher mesh client speeds. Thus, fast deployment for mesh nodes (either clients or routers) needs a metric to measure the prolonged life for the neighbor mesh node at this power level. Power represents heart of a mesh node in an incident area: it is required in order to work for a sufficient length of time in the mesh incident area network with less overhead. At the beginning, the routing overhead for both standard AODV and SM_AODV increases quickly with the increase in the mesh client speed to 5m/s. This is because it does not have an intelligent way of routing traffic through the multi-homed mesh nodes based on power and node-type awareness. “Fig 14” shows that OSPF reduces the routing overheads to a minimum compared to SM_AODV. This is attributed to the ability of OSPF to use channel diversity, without the need to frequently initiate new route discoveries. Furthermore, since OSPF uses a power node-type aware routing metric, routes are established preferentially via mesh routers, resulting in more stable paths.

**Fig 15:**
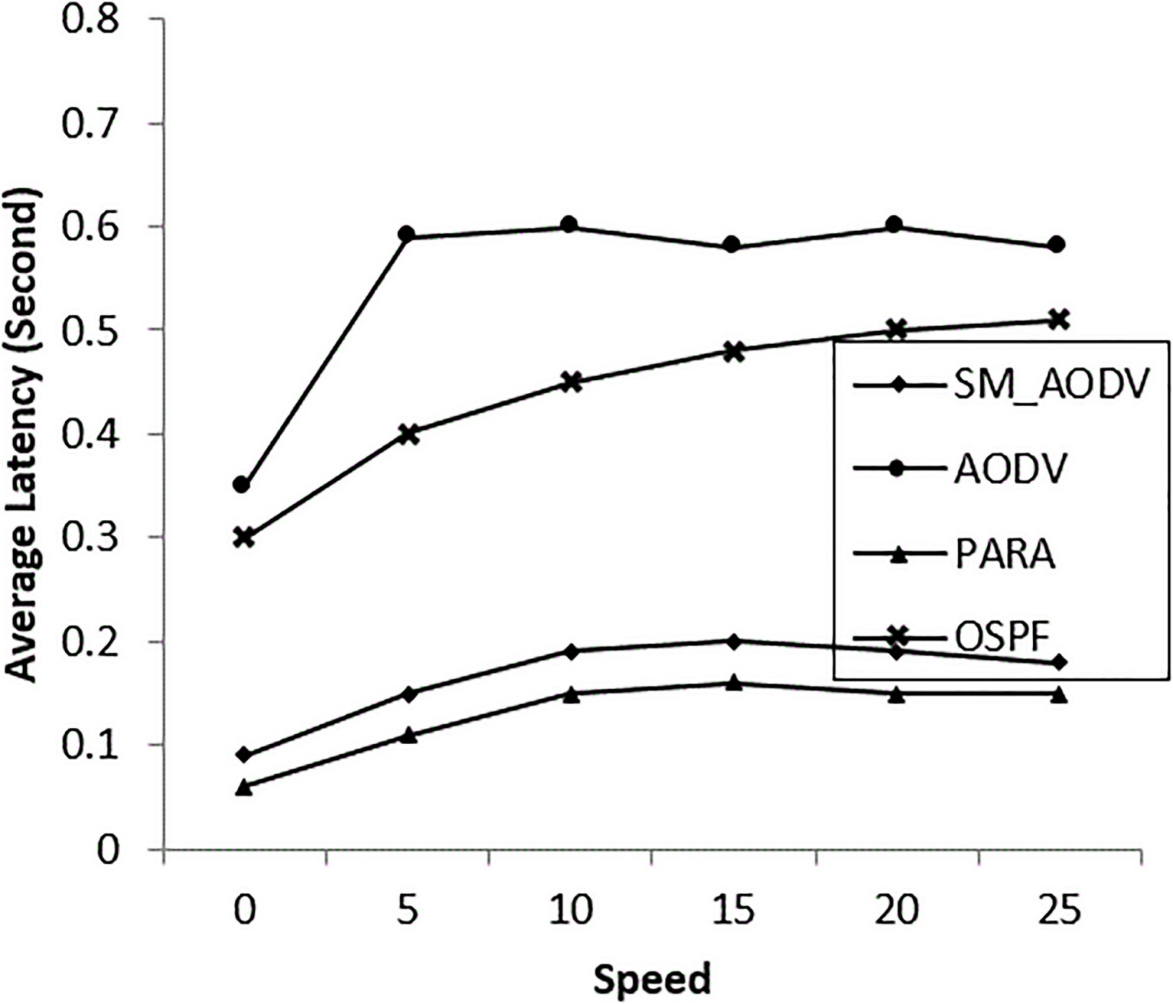
Varying *mesh_client* speeds with (AL).

The important improvement of OSPF over the AODV and SM_AODV is in terms of average packet latency, as shown in “Fig 15”. When multiple data flows are sent via a single wireless interface, the result is extensive contention for the physical medium. So OSPF reduces the medium contention using a channel diversity algorithm “Fig 9”, resulting in more multiple links being available.

### • Scenario 2: Varying the number of *Mesh_ Routers*

**Fig 16:**
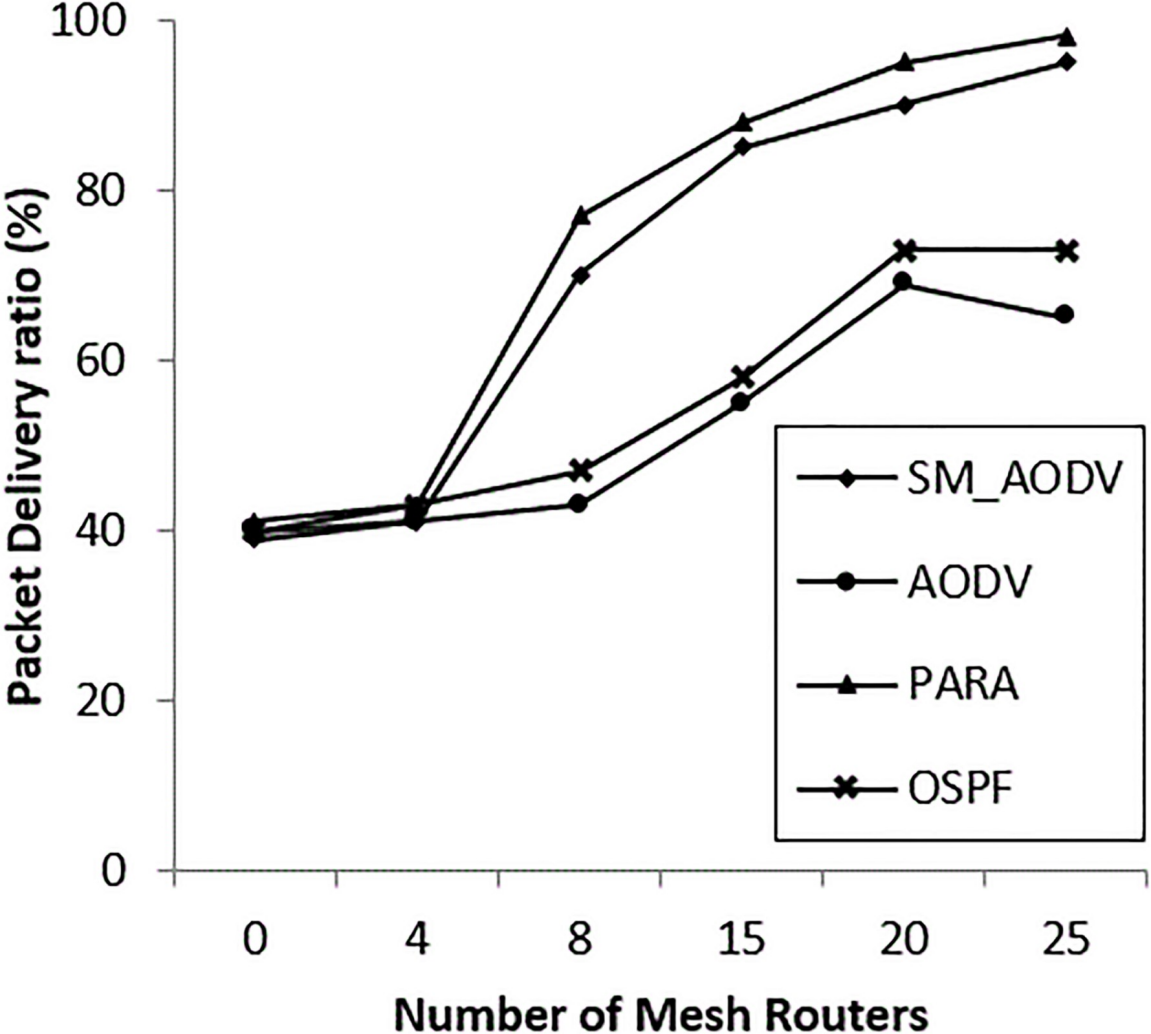
Varying number of *mesh_ routers* with (PDR).

**Fig 17:**
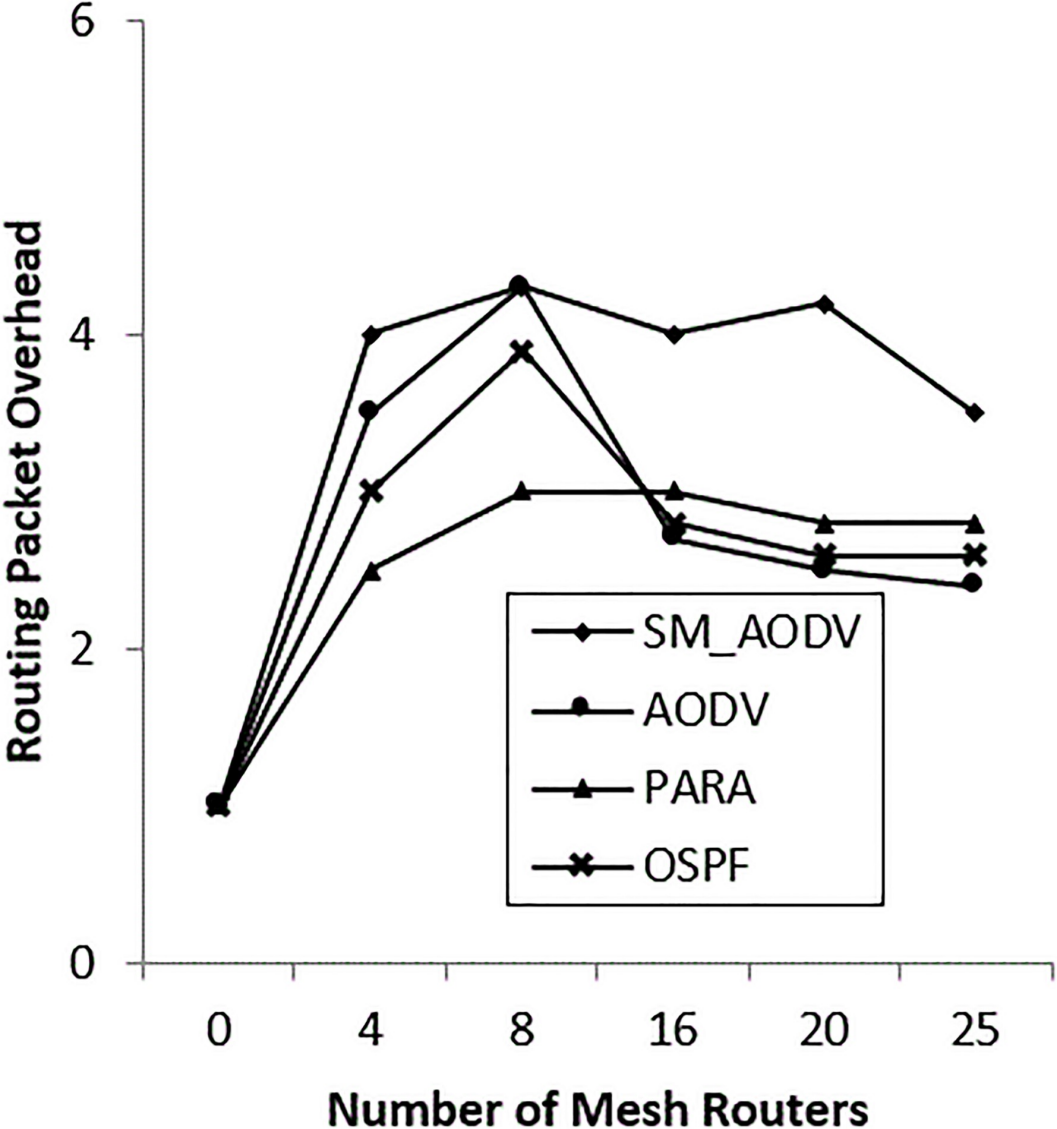
Varying number of *mesh_ routers* with (PRO).

**Fig 18:**
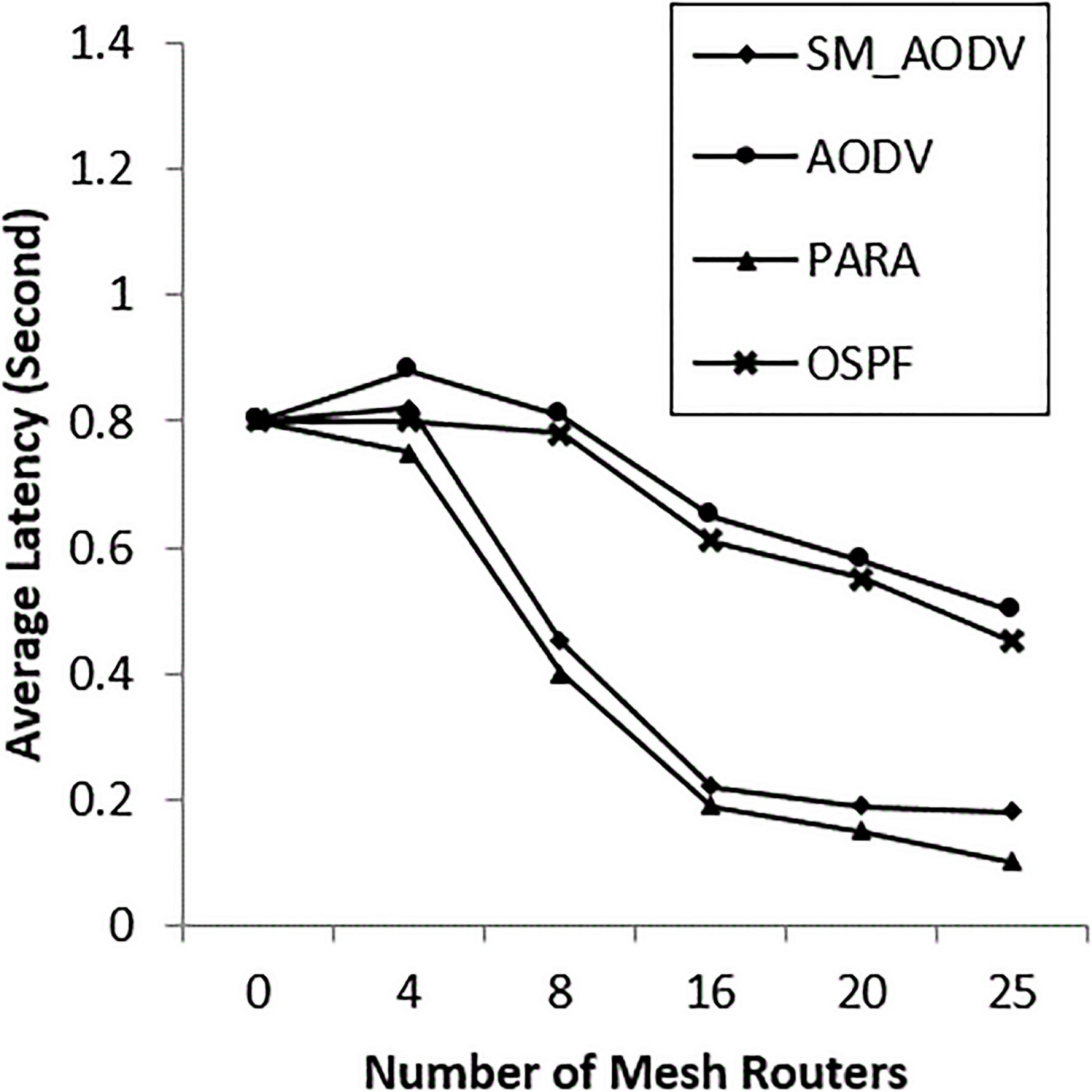
Varying number of *mesh_ routers* with (AL).

The number of mesh routers in the network is varied, and the scenario is considered with n = 0, 4, 8, 15, 20 and 25 mesh routers. The OSPF falls back to standard if there are no mesh routers in the network. “Fig 16” shows a similar PDR for both SM_AODV and OSPF. However, as soon as the number of mesh routers is increased, the PDR of OSPF improves quickly compared with AODV and SM_AODV. This is due to the fact that OSPF is able to take full advantage of the availability of intermediate mesh routers, including the power node-type aware algorithm’s metric, which is not the case for AODV and SM_AODV. When the number of mesh routers is zero, no channel diversity is possible, whereas when the mesh clients are all single-radio devices operating on the same channel, this leads to increasing the contention for the wireless medium. This means falling back to using the mesh clients with conditions of limited channel diversity; resulting in a lower PDR being achieved. Increasing the number of mesh routers improves the performance by increasing the PDR and decreasing both the routing overhead and latency, as shown in “Figs 16–18” respectively, with better results in the OSPF case, SM_AODV and AODV respectively.

OSPF is able to create routes via mesh routers due to its ability to differentiate between node types and power level for each mesh node. This in turn gives the OSPF protocol the ability to dynamically balance the traffic load across the plentiful of available links between neighbouring mesh routers.

AODV and SM_AODV generate more routing overhead at the beginning, with an increase in mesh routers, as shown in “Fig 17”. This is due to frequent route outage and lifetime, interruptions, relocation and breakages, which require repeated route discoveries. The control packet overhead (which is computed as the ratio between the number of control packets generated to the number of received data packets) increases with the increase in the number of mesh routers. This is because the number of multi-radio mesh routers increases in the incident area network.

“Fig 18” shows that, without mesh routers present in the mesh incident area network, the latency of all protocols lies between 750 ms and 900 ms. However, when the number of mesh routers is increased, the latency drops back to 450 ms in the case of AODV, 100 ms in the case of SM_AODV and less than 100 ms for OSPF protocol. This is because OSPF exploits the availability of mesh routers in the network to increase the chance of diverse routes being selected in the channel.

### • Scenario 3: Varying the speed of the *Mesh_ Routers*

**Fig 19:**
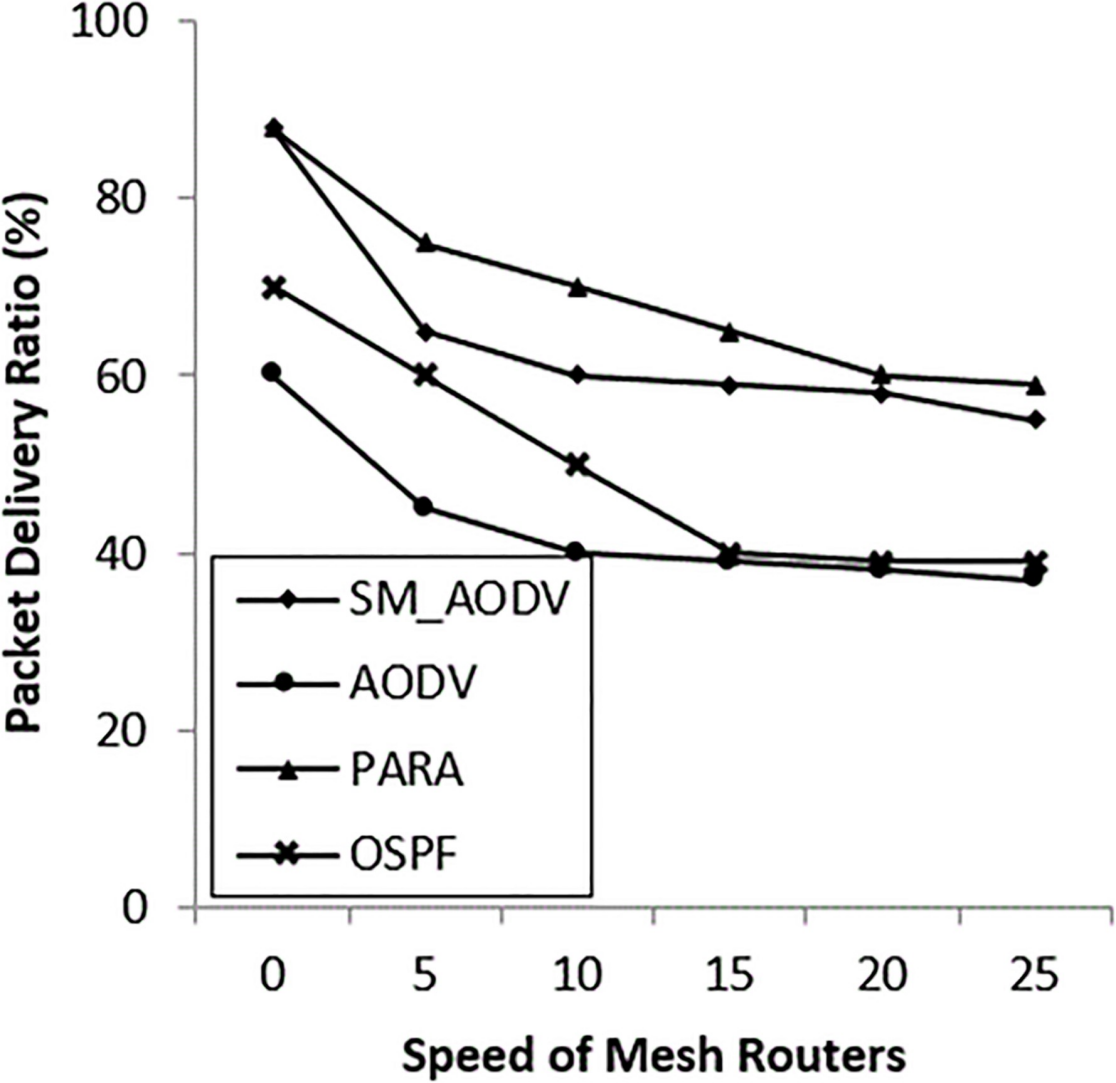
Varying mesh*_ routers speeds* with (PDR).

**Fig 20:**
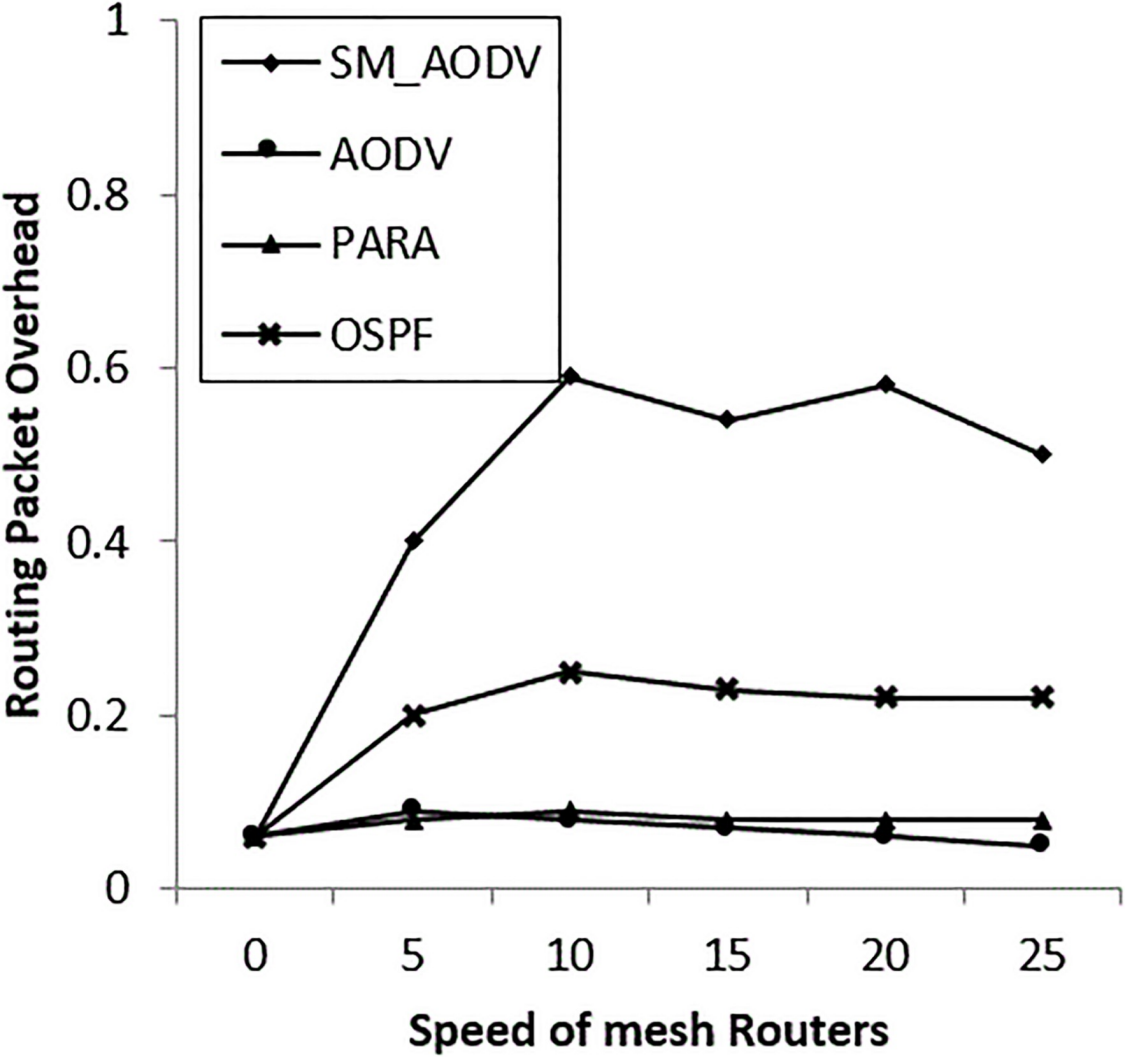
Varying *mesh_ routers speeds with* (PRO).

**Fig 21:**
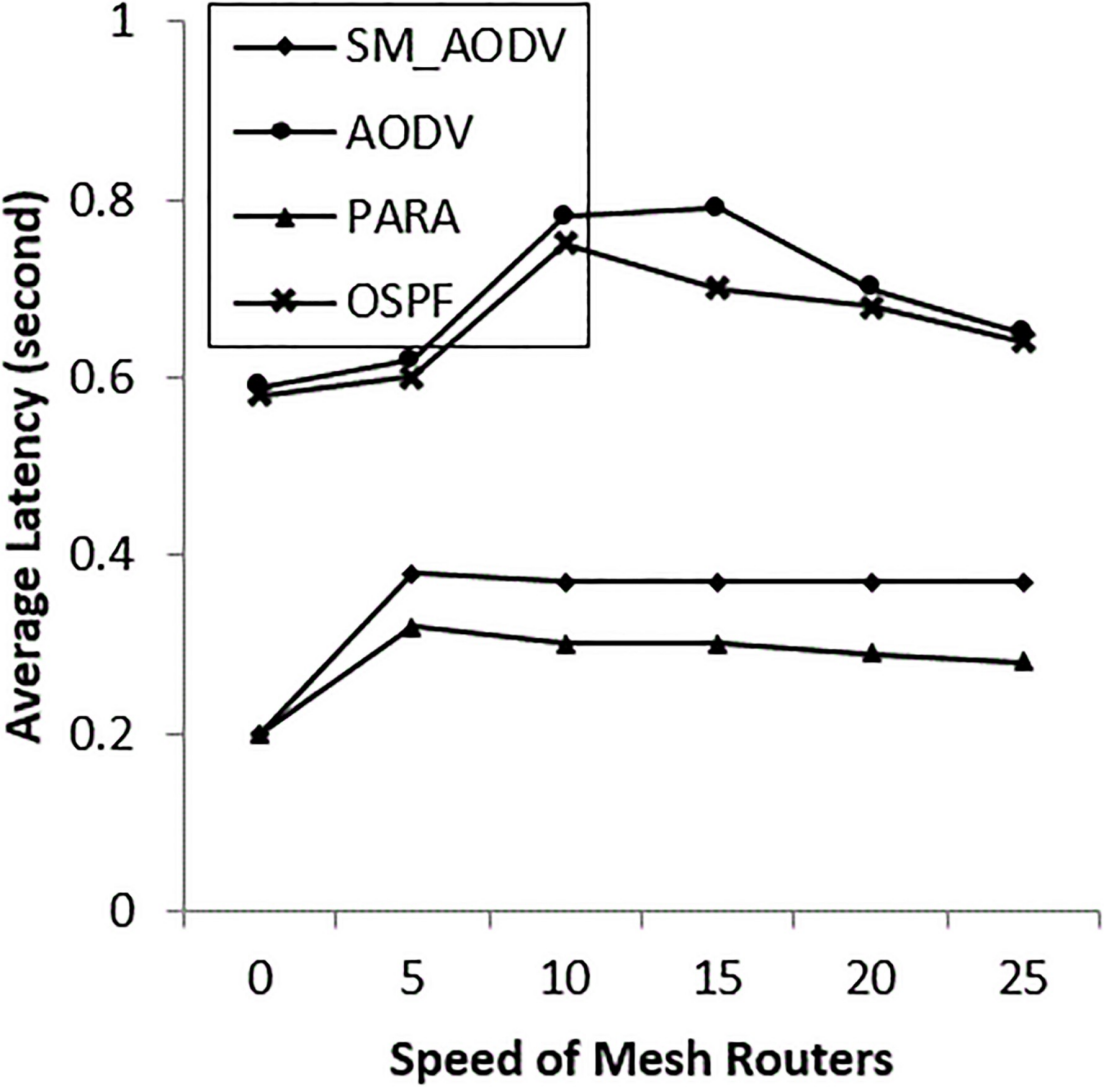
Varying *mesh_ routers speeds* with (AL).

The case of mesh routers being mobile is considered in this scenario, using the same random waypoint mobility model as for the mesh clients. In most situations the routers can be movable, at speeds varying between 0 m/s to 25 m/s. In emergency situations the mesh routers can be mounted on first responder vehicles, or carried by a first responder personal operator (which is our case) at the incident area. The incident area is a disaster area, so it has restrictions and unpredictable obstacles in the environment, such as streets’ lines being disrupted, speed resistance and locations. These restrictions and obstacles degrade network performance. The results in “Figs 19–21” indicate that the OSPF achieves 90% PDR at the beginning and then gradually falls back to 75%, with low packet overhead compared with SM_AODV. The OSPF improves latency, which keeps steady at 300m/s. In general, OSPF outperforms the others, in all respects, due to the fact that, it is able to route packets via stationary mesh routers. This is because the node-type aware metric is employed. Furthermore, the power-aware metric and channel diversity algorithms are used in the incident network, resulting in more stable routes.

### C. Power consumption evaluation in all scenarios

**Fig 22:**
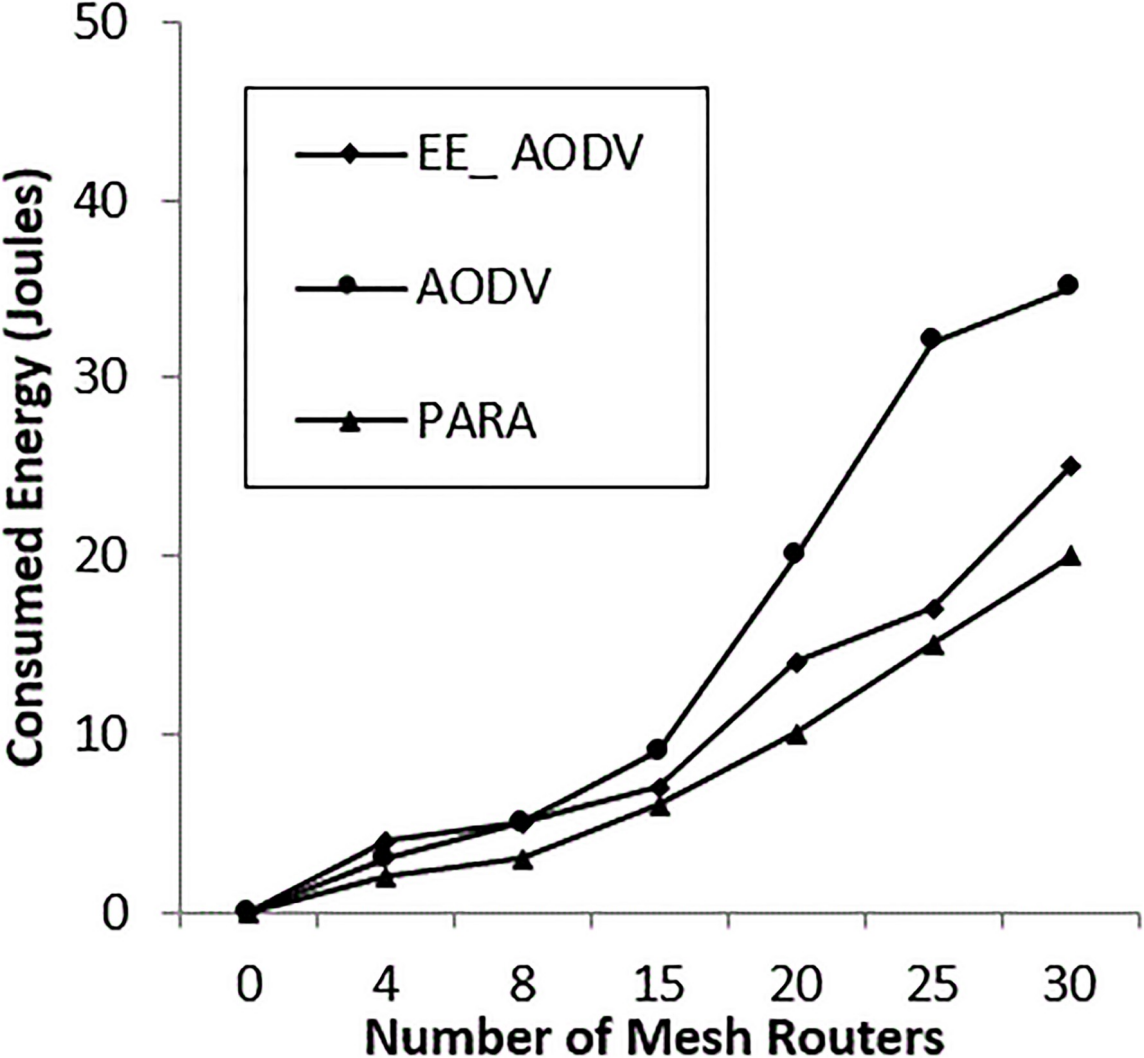
Power consumption for mesh routers.

**Fig 23:**
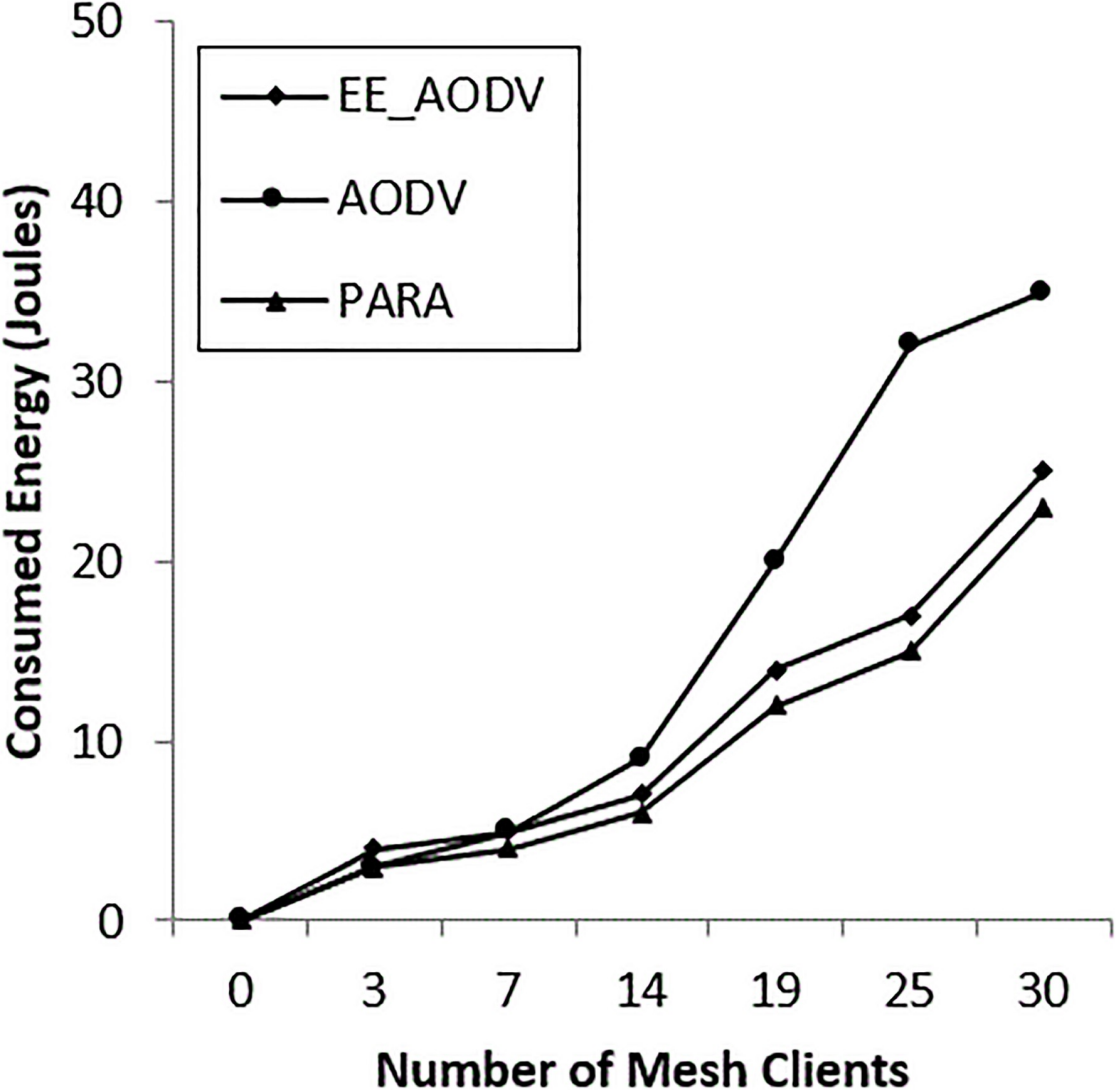
Energy consumption for mesh clients.

The power consumption was evaluated by comparing the PARA algorithm and an energy-efficient scheme using AODV [23]. Scenarios were implemented by varying the number of mesh routers and clients. “Fig 22” shows that, increasing the number of mesh routers consumes less power in the OSPF compared with energy-efficient (EE_AODV) and AODV, with percentages of 20%, 25% and 35%, respectively, at maximum number of mesh routers. In contrast, “Fig 23” shows that the power consumed by increasing the number of mesh clients reaches 23% in the case of OSPF, 27% in the case of EM_AODV and 37% in the case of AODV, at the maximum number of mesh clients.

In both “Figs 22 and 23”, it can be seen that AODV is consuming a great deal of power. This is because it is generating more control packets to discover and establish routes. Furthermore, it lacks intelligent algorithms, such as channel diversity, node-type aware and power aware. In contrast, OSPF saves power by an average of up to 86%. this is because it is exploiting channel diversity, and using power aware, and node-type aware algorithms to discover and establish routes through the incident area network. The power node-type aware algorithm metric is measuring power utilization for the next node, with mesh routers being preferable. The availability of mesh router nodes in the incident area network is recommended, due to their intelligent ability to route the packets to the required interface in the first instance. However, increasing the number of mesh client nodes is undesirable in incident area networks (IANs). Thus, the PARA algorithm balances the number of mesh node clients against the number of mesh node routers. It uses α and β weight values based on network size and the total number of nodes which contribute in the incident area network. These values are assumed as α = 4 and β = 1, so to balance mesh routers and mesh clients, based on Eq 5, either the number of mesh routers is increased (this consumes more power) or the value of β is inverted to be (1β) within the incident area network. It should be borne in mind that the incident area network has restrictions, such as the device’s location and power charging resources and street lines, and obstacles, such as building locations and collapsed monument.

The PARA approach reduces power wastage by reducing the volume of routing control packets, compared with EE_AODV and AODV, which waste power in finding the routing path on demand.

**Fig 24:**
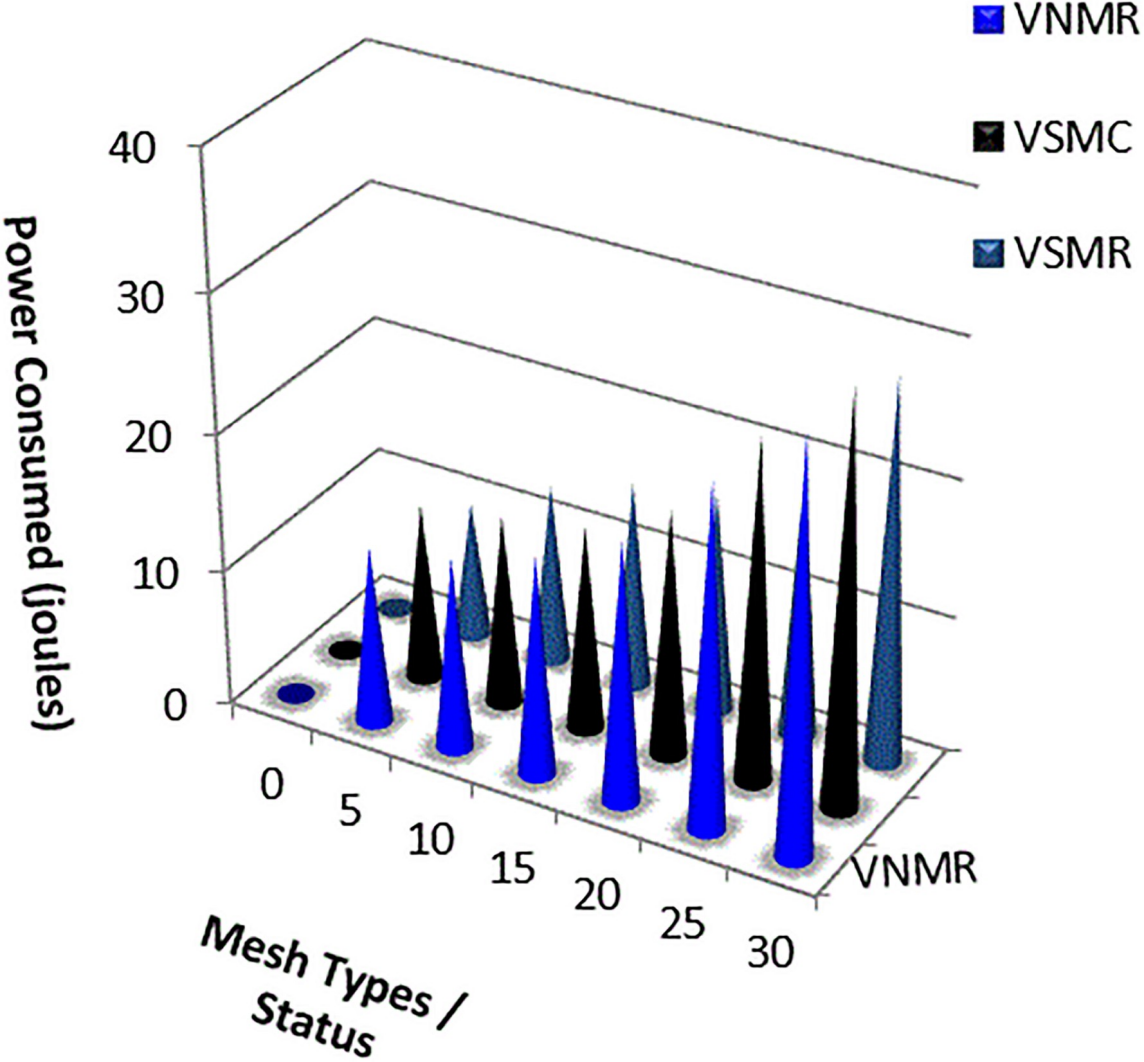
Power consumptions using PARA.

Obviously, the mesh routers consume less power than the mesh clients do, because a mesh client is mostly mobile, with fast deployment, and is sending and receiving, and generating more routing control packets, and less capacity. The power consumption was evaluated by comparing the three scenarios above, as shown in “Fig 24”. The average power consumption percentages for each scenario (varying the speed of *Mesh_Client* (VSMC), varying the number of *Mesh_ Router* (VNMR), and varying the speed of *Mesh_ Router* (VSMR)) were 18%, 18%, and 15%, respectively. Varying the numbers of mesh routers consumed greater power, because the nodes generated redundant routing control packets, to discover/establish new paths through an unstable area such as an incident area. Speed resistance is expected in the environmental conditions of an incident area. Varying the speed of the mesh client and mesh routers consumes less power than increasing the number of mesh nodes (either clients or routers). This reduction in power is observed under all scenarios (i.e. mobility patterns, traffic patterns, network size and area layout). Additionally, this reduction in power is observed under all restrictions of the incident area network. Generally, this reduction in power consumption is due to the reduction in the number of routing control packets.

## Conclusions

This paper has proposed a power and node-type aware routing algorithm which exploits awareness of the power level and type of mesh node to find the optimal route to the final destination. Three scenarios were used to evaluate the performance of the PARA algorithm under varying the mobility and traffic load conditions with different node configurations, and the results were compared with those of OSPF, SM_AODV and AODV. The simulation results demonstrated that the Power Aware Routing Algorithm (PARA) prolongs the network routes’ connectivity and optimizes the network’s performance. In most scenarios, the Packet Delivery Ratio (PDR) is almost 100%, and the average latency was found to be less than 100 ms. In addition, a significant enhancement was obtained in minimizing the power consumption by giving priority to mesh routers. These routers receive and forward the packets intelligently in the incident area network, which achieves a good power saving. PARA reduced the percentages of power used by 82%, 82%, and 85%, for all scenarios, i.e., when varying the speeds of Mesh_Client (VSMC), the number of Mesh_ Router (VNMRs), and the speeds of Mesh_ Router (VSMR), respectively. The main strength of the proposed algorithm is to optimize the network performance in terms of the power saving by reducing the number of routing control packets. However, further investigation needs to be carried out in order to optimize routing algorithms over multiple hops within wireless mesh networks, by integrating different kind of networks, such as Digital Video Broadcasting (DVB)-Satellite, wireless sensor network and cloud computing networks, achieving complementary systems during emergency situations. It is intended to carry out further investigation to minimize the power consumption using different routing metrics to be used in Internet of Things (IoT) devices.
